# Multimodal plasma and urinary cell-free DNA profiling improves risk stratification in newly diagnosed prostate cancer

**DOI:** 10.1038/s41698-026-01343-y

**Published:** 2026-03-02

**Authors:** Anja Lisa Riediger, Samaneh Eickelschulte, Florian Janke, Daniela Janscho, Olga Lazareva, Daniel Hübschmann, Stefan Duensing, Oliver Stegle, Holger Sültmann, Magdalena Görtz

**Affiliations:** 1 Junior Clinical Cooperation Unit, Multiparametric Methods for Early Detection of Prostate Cancer, German Cancer Research Center (DKFZ), Heidelberg, Germany; 2 Department of Urology, University Hospital Heidelberg, Heidelberg, Germany; 3 Division of Cancer Genome Research, German Cancer Research Center (DKFZ), German Cancer Consortium (DKTK), and National Center for Tumor Diseases (NCT), Heidelberg, Germany; 4 Heidelberg University, Faculty of Biosciences, Heidelberg, Germany; 5 Division of Computational Genomics and Systems Genetics, German Cancer Research Center (DKFZ), Heidelberg, Germany; 6 Genome Biology Unit, European Molecular Biology Laboratory (EMBL), Heidelberg, Germany; 7 Molecular Precision Oncology Program, National Center for Tumor Diseases (NCT), Heidelberg, Germany; 8 Heidelberg Institute of Stem Cell Technology and Experimental Medicine (HI-STEM), Heidelberg, Germany; 9 German Cancer Consortium (DKTK), Heidelberg, Germany; 10 Molecular Urooncology, Department of Urology, University Hospital Heidelberg, Heidelberg, Germany

**Keywords:** Biomarkers, Cancer, Computational biology and bioinformatics, Genetics, Oncology

## Abstract

Prostate cancer (PCa) is a heterogeneous disease, impeding early detection and risk stratification. Liquid biopsies (LBx) enable minimally invasive tumor profiling, but circulating tumor-derived DNA (ctDNA) detection remains difficult, particularly in early-stage PCa. We developed a multimodal LBx approach combining genomic and epigenomic cell-free DNA (cfDNA) features in plasma and urine from newly diagnosed PCa patients to improve early characterization of PCa and risk stratification of aggressive disease. Plasma and urine samples from 55 localized PCa (lPCa) patients, 18 advanced PCa (aPCa) patients, and 36 cancer-free controls were subjected to low-coverage whole-genome sequencing and methylated DNA immunoprecipitation sequencing to assess fragmentation, chromosomal instability, and methylation in cfDNA. This complementary approach yielded a 45% ctDNA detection rate in newly diagnosed PCa. Major differences were observed between aPCa and controls, reflecting increasing signals with tumor progression. Epigenomic cfDNA features differentiated lPCa from aPCa, and ctDNA was detected in 46% of PCa patients with prostate-specific antigen <10 ng/mL, suggesting potential for risk stratification. This study highlights the value of multimodal LBx approaches for early characterization of primary PCa and identification of aggressive disease at initial diagnosis. Integration into clinical workflows could complement diagnostics and support personalized decision-making tailored to patients’ PCa risk profiles.

## Introduction

Prostate cancer (PCa) is a clinically and molecularly heterogeneous disease, presenting major challenges for early detection, risk stratification, and personalized treatment decisions^1,2^.

Current diagnostics, including prostate-specific antigen (PSA) testing, multiparametric magnetic resonance imaging (mpMRI), and invasive prostate tissue biopsy, provide limited insight into tumor heterogeneity^3^, impeding precise risk stratification. A major challenge is distinguishing clinically significant PCa^4,5^, which requires aggressive treatment, from indolent disease, in order to avoid overtreatment of low-risk cancers unlikely to cause harm^6^. PSA remains a widely used screening biomarker, but its limited specificity –especially in the intermediate “gray zone” of 4–10 ng/mL^7^– can also lead to false-positive results and unnecessary biopsies^8^. To address these limitations, major urological guidelines recommend integrating risk calculators into the diagnostic pathway^5,9^. However, the distinction between clinically significant and insignificant PCa remains imprecise and lacks universally applied criteria^4,5^. This highlights the need for novel biomarkers that could improve diagnostic accuracy, enabling early identification of aggressive disease and supporting patient-tailored management. Liquid biopsy (LBx) facilitates molecular characterization of the tumor and its metastases, allowing for minimally invasive disease and therapy monitoring^10,11^. The analysis of cell-free DNA (cfDNA) is a well-established approach to detect genomic alterations, indicating the presence of circulating tumor DNA (ctDNA) and enabling tumor burden estimation^12^. Genomic ctDNA detection in PCa is challenging, due to overall limited ctDNA shedding and low mutational burden^13,14^. Epigenomic profiling holds promise for early tumor characterization, given the early occurrence and tissue specificity of epigenomic changes, such as DNA methylation^15,16^. CfDNA fragmentation patterns were equally shown to harbor tumor-specific information and reflect ctDNA levels^17–19^. This study developed a multimodal LBx approach, analyzing genomic and epigenomic cfDNA features with low-coverage whole-genome sequencing (lcWGS) and cell-free methylated DNA immunoprecipitation sequencing (cfMeDIP-seq) in plasma and urine of newly diagnosed PCa patients and individuals without cancer. The complementary analysis aimed to improve ctDNA detection, supporting tumor characterization and early risk stratification of aggressive disease.

## Results

### Patient characteristics and next-generation sequencing

Seventy-three patients with newly diagnosed PCa without any prior treatment, and 36 cancer-free controls were enrolled (Table 1). The majority (75%) had localized PCa (lPCa, N0 M0), while 25% presented with lymph node (N1) and/or distant metastases (M1), referred to as advanced PCa (aPCa). PCa patients had significantly higher PSA levels compared to controls (median: 8.1 ng/ml vs. 1.95 ng/mL, *p* = 4.1e−08), with advanced PCa showing the highest levels (median: 18.8 ng/mL, *p* = 0.015; Table 1). LcWGS and (cf)MeDIP-seq were performed on 109 plasma and 102 urine samples (Table 1), as well as on PCa tissue and matched buffy coat samples from eight PCa patients. Plasma was available for all, but urine was missing for five men. LcWGS libraries yielded a median of 72 million (M) quality-filtered total reads (range 36–110 M) in LBx samples and 62 M total reads (range 56–71 M) in tissue/buffy coat samples, resulting in a median genome-wide coverage of 2.76× (range 1.38–4.28×) and 2.32× (range 2.07–2.66×), respectively (Supplementary Table S1). CfMeDIP-seq libraries yielded a median of 60 M total reads (range 0.053–137 M) in LBx samples and 54 M total reads (range 34–80 M) in tissue/buffy coat samples, enriching for DNA fragments containing 5-methylcytosine (5mC). Across samples, a median of 79.1% and 76.1% of all genomic CpG sites were covered by at least one read in LBx and tissue/buffy coat samples, respectively. Among the captured CpG sites, 36.0% and 35.9% were covered by more than five reads (Supplementary Table S1). One urine sample was excluded due to low sequencing depth.

**Table 1:** Overview of patients and LBx samples

PCa, localized disease (stage I-III)		(*n* = 55)
age, median (range)		66 (49–80)
PSA level (ng/mL), median (range)		7.7 (2.7–40.0)
	**plasma**	**urine**
low risk	4^‡^	3^‡^
GS 6 (*n* = 4)	4	3
Intermediate risk	42	38*
GS 7a (*n* = 37)	37	33*
GS 7b (*n* = 5)	5	5
high-risk	9	9
GS 7a/7b (*n* = 2)	2	2
GS 8 (*n* = 1)	1	1
GS 9 (*n* = 6)	6	6

PCa, disseminated disease (stage IV)		(*n* = 18)
age, median (range)		65 (58–75)
PSA level (ng/mL), median (range)		18.8 (4.1–249.0)
	**plasma**	**urine**
lymph node metastases, N1 M0	9	9
GS 7a/7b (*n* = 4)	4	4
GS 8 (*n* = 1)	1	1
GS 9 (*n* = 4)	4	4
distant metastases, M1	9	9
GS 7a/7b (*n* = 3)	3	3
GS 8 (*n* = 3)	3	3
GS 9 (*n* = 2)	2	2
GS 10 (*n* = 1)	1	1

control cohort		(*n* = 36)
age, median (range)		58 (37–74)
PSA level (ng/mL), median (range)		1.95 (0.26–13.1)
	**plasma**	**urine**
controls with PSA < 2 ng/mL (*n* = 20)	20	20
controls with PSA > 2 ng/mL (*n* = 16) no evidence of malignancy (MRI, biopsy)	16	15

PCa stages were determined based on UICC (stage I-IV)^68^ and D’Amico Risk Classification^69^.

*LBx* Liquid Biopsy, *M0/M1* presence/absence of distant metastases, *MRI* magnetic resonance imaging, *N1* presence of lymph node metastases, *GS* Gleason Score, *NA* data not available, *n* number, *PCa* prostate cancer, *PSA* prostate-specific antigen, *UICC* Union for International Cancer control, ^‡^ one patient had additional sample collection after one year with upgrade to PCa GS 7b, *one sample excluded after sequencing quality control.

LcWGS and cfMeDIP-seq data were employed to evaluate genome-wide chromosomal instability, structural alterations, and methylation, as well as fragmentation patterns of plasma and urinary cfDNA. For each analysis type, selected LBx features were used to detect ctDNA in samples from tumor patients, as detailed in the following sections. The ctDNA detectability threshold was set at the 95th or 5th percentile of control samples for markers increased or decreased in tumor patients, respectively, and samples with values beyond these thresholds were classified as ctDNA-positive.

### CfDNA methylation patterns distinguished metastatic from non-metastatic PCa and controls

**Fig. 1:**
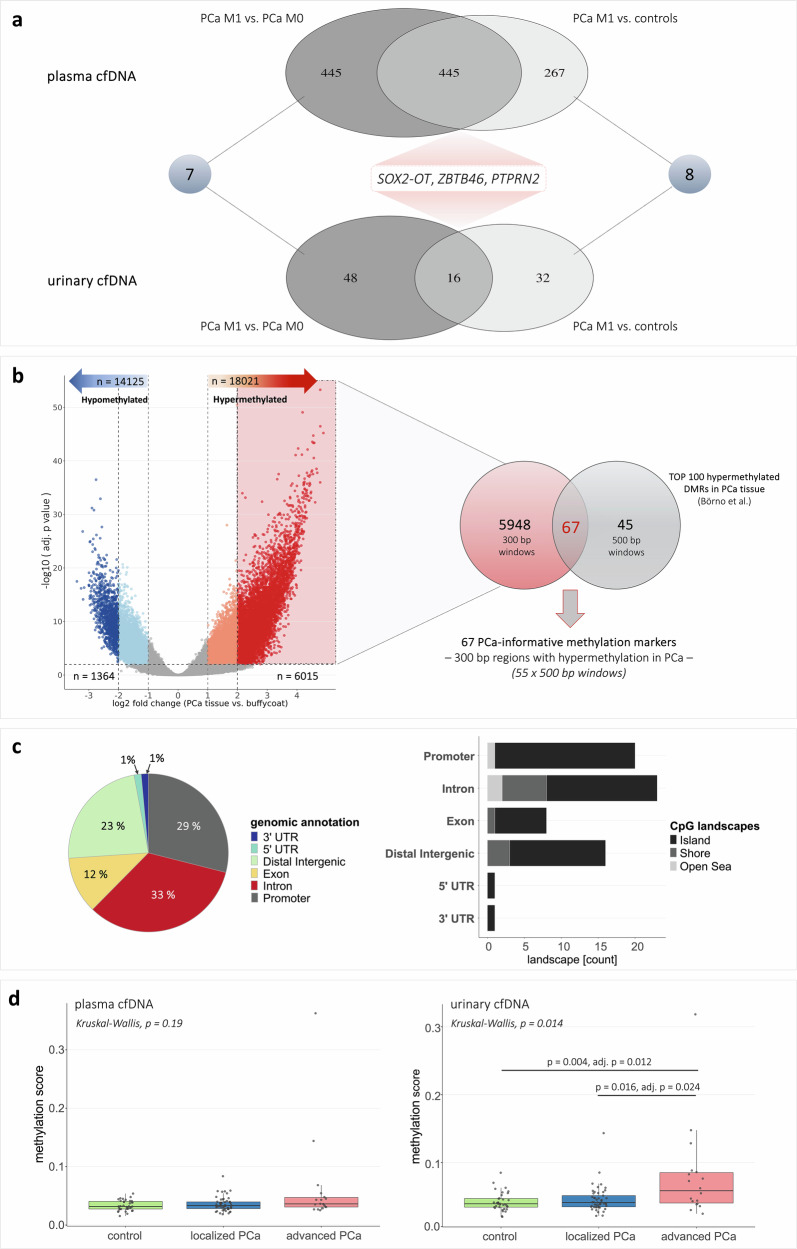
Genome-wide methylation profiling in PCa tissue and LBx samples. **a** Results from the determination of DMRs between mPCa patients and non-mPCa patients or controls, respectively, in plasma and urinary cfDNA. **b** Determination of DMRs between PCa tissue and matched buffy coat samples revealed significant (adjusted *p* < 0.01), hyper- and hypomethylated regions in PCa tissue. Identified 6015 hypermethylated regions (300 bp windows) with log2 fold change > 2 in PCa tissue compared to buffy coat were overlapped with 100 top-ranked, hypermethylated DMRs (500 bp windows) obtained from an external data set (Börno et al. ^20^), and 67 common regions (300 bp windows) were identified. Tissue-informed methylation markers were applied to cfMeDIP-seq data from LBx samples. **c** Left: Genomic annotation of 67 methylation marker regions; location within 3’ or 5’UTR, distal intergenic region, downstream region, exon, intron, or promoter region. Right: Genomic annotation and assessment of CpG-associated landscapes (island, open sea, shelf, shore) within the 67 methylation marker regions. **d** Methylation scores (median of beta-values in 67 methylation marker regions) in plasma cfDNA (left) or urinary cfDNA (right) from cancer-free controls, lPCa patients, and aPCa patients. Each dot represents one sample. Box plot center lines indicate the median, and boxes illustrate the interquartile range with Tukey whiskers. The three cohorts were compared using Kruskal-Wallis testing, followed by Dunn’s post hoc test. Only significant differences are shown. adj. p adjusted *p* value, aPCa advanced prostate cancer, bp base pair, cfDNA cell-free DNA, DMR differentially methylated region, LBx Liquid Biopsy, lPCa localized prostate cancer, M0 / M1 absence/presence of distant metastases, mPCa metastatic prostate cancer, *n* number, PCa prostate cancer, PTPRN2 protein tyrosine phosphatase receptor type N2, SOX2-OT SOX2 overlapping transcript, UTR untranslated region, ZBTB46 zinc finger and BTB domain containing 46.

Genome-wide methylation profiling in LBx was conducted to explore cfDNA methylation differences across PCa groups and controls. Differential analysis revealed no significant differentially methylated regions (DMRs) between all PCa samples and controls (Supplementary Table S2). However, PCa patients with distant metastases (metastatic PCa, mPCa) showed distinct methylation patterns in both plasma and urine compared to controls and PCa patients without distant metastases (non-mPCa), particularly in cases where ctDNA was also detected via genomic analysis. In plasma cfDNA, 712 DMRs were identified in mPCa compared to controls, and 890 DMRs in mPCa vs. non-mPCa (Fig. 1a), with most regions showing hypermethylation in mPCa. Overlapping these sets revealed 445 shared DMRs, including 392 hypermethylated and 21 hypomethylated regions in mPCa (Supplementary Table S2*+* Supplementary Fig. S1). Hypermethylated regions were found in distal intergenic regions (31%), introns (25%), and promoters (23%; Supplementary Fig. S1). In urinary cfDNA, only a few DMRs were identified: 48 in mPCa vs. controls and 64 in mPCa vs. non-mPCa, with 16 overlapping regions (13x hypermethylated, 3x hypomethylated), primarily located in introns (50%) and exons (25%; Fig. 1a; Supplementary Fig. S1). Comparison of DMRs in plasma and urine revealed eight shared regions in mPCa vs. controls, and seven in mPCa vs. non-mPCa, including three identical hypermethylated regions in both comparisons, associated with the genes *SOX2-OT*, *ZBTB46*, and *PTPRN2* (Fig. 1a). The cfDNA-derived DMRs were analyzed to explore methylation patterns associated with tumor aggressiveness, but were not used for ctDNA detection, which was based on DMRs identified in PCa tissue and validated using an independent dataset, described in the following section.

### PCa tissue methylation markers were elevated in LBx from PCa patients

Genome-wide differential methylation analysis was performed in PCa tissue and matched buffy coats to identify PCa-specific methylation patterns. Overall, 32,146 significant DMRs were determined, including 6015 hypermethylated (log fold changes (logFC) > 2) and 1364 hypomethylated (logFC < -2) regions (Fig. 1b; Supplementary Fig. S2). Most were located in introns and distal intergenic regions. Promoters accounted for 13% of hypermethylated and 6% of hypomethylated DMRs.

The 6015 hypermethylated DMRs were compared to a published MEDIP-seq dataset^20^, reporting 100 top-ranked, hypermethylated DMRs between 51 primary PCa and 53 normal prostate tissue samples. This comparative analysis validated the internal findings against an independent dataset and highlighted the most consistent, PCa–specific methylation regions across cohorts. These overlapping, most informative regions were subsequently applied to the LBx samples to assess cfDNA methylation levels and ctDNA detectability. Sixty-seven shared regions were identified (Fig. 1b), predominantly located in introns (33%) and promoters (29%), with methylated CpGs mainly occurring inside CpG islands (Fig. 1c). Three intronic regions were associated with *PTPRN2*.

Further validation using an external cfMeDIP-seq dataset of 133 plasma samples^21^ revealed that metastatic castration-resistant PCa (mCRPC, *n* = 103) clustered separately and demonstrated higher heterogeneity than lPCa (*n* = 30) based on methylation levels in these 67 regions, confirming their relevance as PCa biomarkers, particularly in advanced disease (Supplementary Fig. S3). Principal component analysis (PCA) based on methylation levels in the selected regions and in 67 randomly chosen regions revealed weaker separation between lPCa and mCRPC in the random set, supporting the PCa-specificity of the selected regions (Supplementary Fig. S3). The external dataset comprised two distinct cohorts for which genome-wide molecular differences were expected, though potential confounding factors or batch effects could not be excluded.

The 67 hypermethylated regions were analyzed in our cfMeDIP-seq data, and a synoptic methylation score was calculated, showing an increasing trend from controls to lPCa to aPCa in both plasma and urine (Fig. 1d). Advanced PCa patients harbored significantly higher scores compared to both lPCa patients (*p* = 0.016, adjusted *p* = 0.02) and controls (*p* = 0.004, adjusted *p* = 0.01) in urinary cfDNA, while differences in plasma were not significant. The methylation score of 12 plasma (7x lPCa, 5x aPCa) and 12 urine samples (4x lPCa, 8x aPCa) exceeded the ctDNA detectability threshold. Five patients had elevated scores in matched plasma and urine, with higher levels in urine.

### Complementary genomic profiling in plasma and urine improved ctDNA detection

**Fig. 2:**
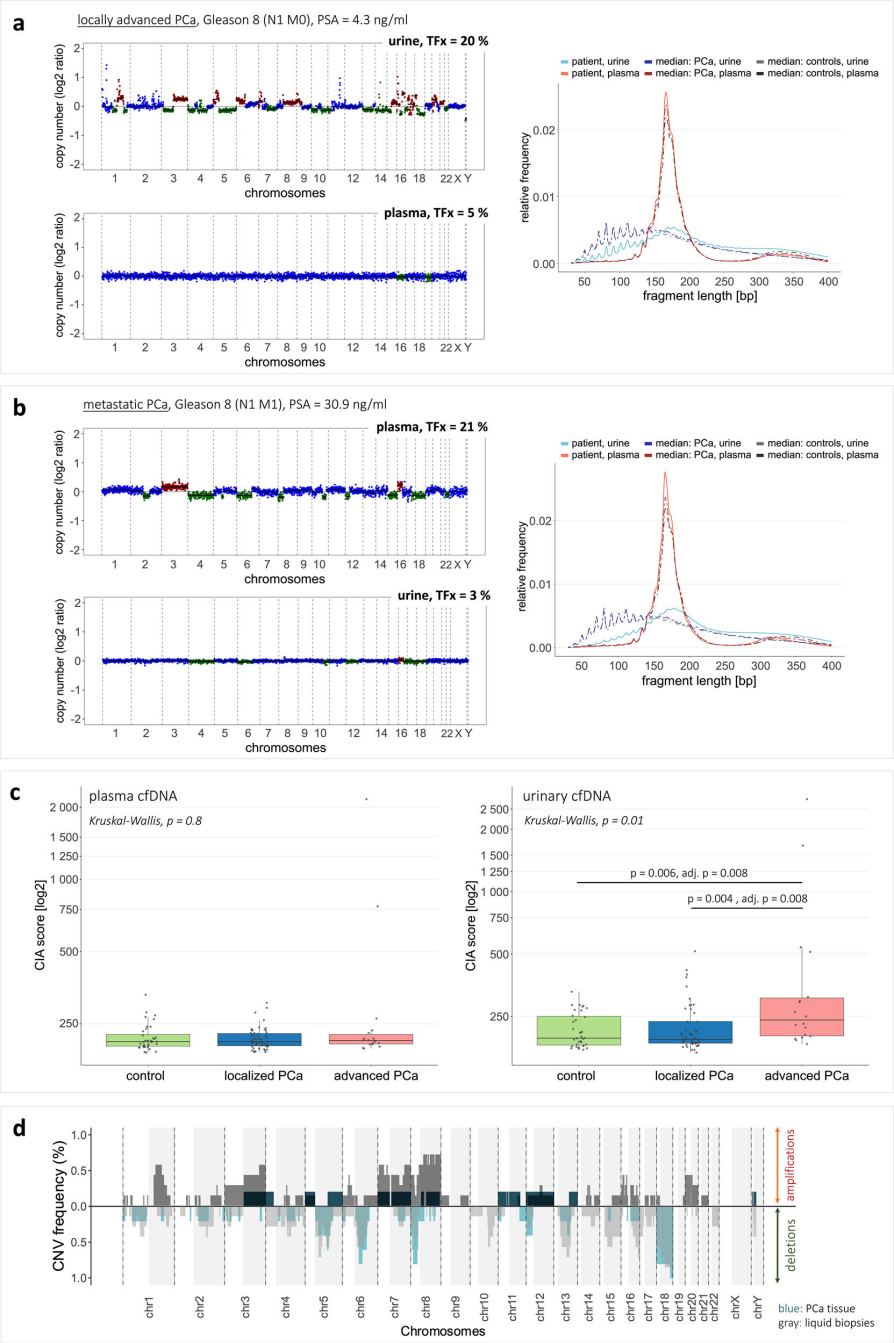
Genomic analysis in plasma and urinary cfDNA. **a** Complementary CNV profiles of plasma (left, bottom) and urine (left, top) samples from one patient with locally advanced PCa. Distinct CNVs were observed in the urine sample, while the plasma sample showed only few alterations. Right: Plasma and urinary cfDNA fragmentation profiles of the patient´s samples differed from the median profiles of all tumor and control samples. **b** Complementary CNV profiles of plasma (left, top) and urine (left, bottom) samples from one patient with mPCa. Distinct CNVs were observed in the plasma sample, while the urine sample showed only few alterations. Right: Plasma and urinary cfDNA fragmentation profiles of the patient´s samples differed from the median profiles of all tumor and control samples. **c** CIA scores in plasma cfDNA (left) or urinary cfDNA (right) from cancer-free controls, lPCa patients, and aPCa patients. Each dot represents one sample. Box plot center lines indicate the median, and boxes illustrate the interquartile range with Tukey whiskers. The three cohorts were compared using Kruskal-Wallis testing, followed by Dunn’s post hoc test. Only significant differences are shown. **d** Summary of recurrent amplifications and deletions in LBx samples (gray color) and eight PCa tissue samples (blue/turquoise color). Only LBx and PCa tissue samples with detectable CNVs and an estimated TFx >10% were considered (PCa tissue: *n* = 5, LBx: *n* = 9). The y-axis indicates the frequency of a detected copy number state at the chromosomal coordinate specified on the x-axis across the samples. Areas shaded in gray represent the q-arm of the respective chromosome. chr chromosome, CIA chromosomal instability analysis, CNV copy number variation, N0/N1 absence/presence of lymph node metastases, TFx tumor fraction.

Genome-wide copy number variations (CNVs) were profiled in plasma and urinary cfDNA via lcWGS, assessing ctDNA presence and estimating the underlying tumor fraction (TFx) in cfDNA. CtDNA was mainly detected in either plasma or urine of the PCa patients, indicating complementary CNV profiles (two examples in Fig. 2a, b). Five plasma samples (3x lPCa, 2x aPCa) and eight urine samples (5x lPCa, 3x aPCa) harbored ctDNA (Supplementary Fig. S4). In-silico size selection aimed to enrich for ctDNA, as previously shown for plasma cfDNA with 90–150 bp length^18,22^, increasing the number of ctDNA-positive plasma samples to nine (5x lPCa, 4x aPCa) and raising TFx across samples, i.e., evident in two mPCa samples (TFx increases +32% and +24%, respectively). In urine, no optimal size selection was identified, due to heterogeneous cfDNA fragmentation (Supplementary Fig. S4).

The chromosomal instability analysis (CIA) score served as an additional genomic biomarker, targeting in particular highly deviating regions to reduce background noise. The CIA score correlated with the estimated TFx in plasma and in urine (Spearman´s ρ = 0.44 and 0.76, respectively; Supplementary Fig. S5). Tumor samples had higher median CIA scores than controls in both fluids, with significant differences seen in urine between aPCa and controls or lPCa, respectively (both adjusted *p* = 0.008; Fig. 2c). Five plasma (3x lPCa, 2x aPCa) and 14 urine samples (7x lPCa, 7x aPCa), including one matched plasma-urine pair, exceeded the detectability threshold, indicating ctDNA presence.

CNV analysis in PCa tissue identified samples with high, low, or no detectable genomic alterations (Supplementary Fig. S6), consistent with previous studies describing CNV-based molecular subgroups in primary PCa according to the extent of genomic instability^2,23^. Five tissue samples exhibited CNVs, with four showing predominantly deletions and one displaying widespread genomic instability with both deletions and amplifications. The remaining three samples showed no detectable CNVs, despite sufficient tumor cell content (40–90%) and large tumor sizes. Comparisons between CNV-positive tissue samples and matched plasma and urine were inconclusive, as all LBx samples had TFx below the ctDNA detectability threshold.

LBx samples with TFx >10% harbored recurrent genomic alterations, consistent with our findings in eight PCa tissue samples and known alterations in primary PCa^2,23^. Deletions in 5q, 6q, 8p, 13q, and 18 were present in >40% of both tissue and LBx samples; gains in one tissue sample (3q, 5p, 7, 8q) were found in up to 60% of positive LBx samples (Fig. 2d).

### Plasma and urinary cfDNA fragmentation differed between PCa patients and controls

**Fig. 3:**
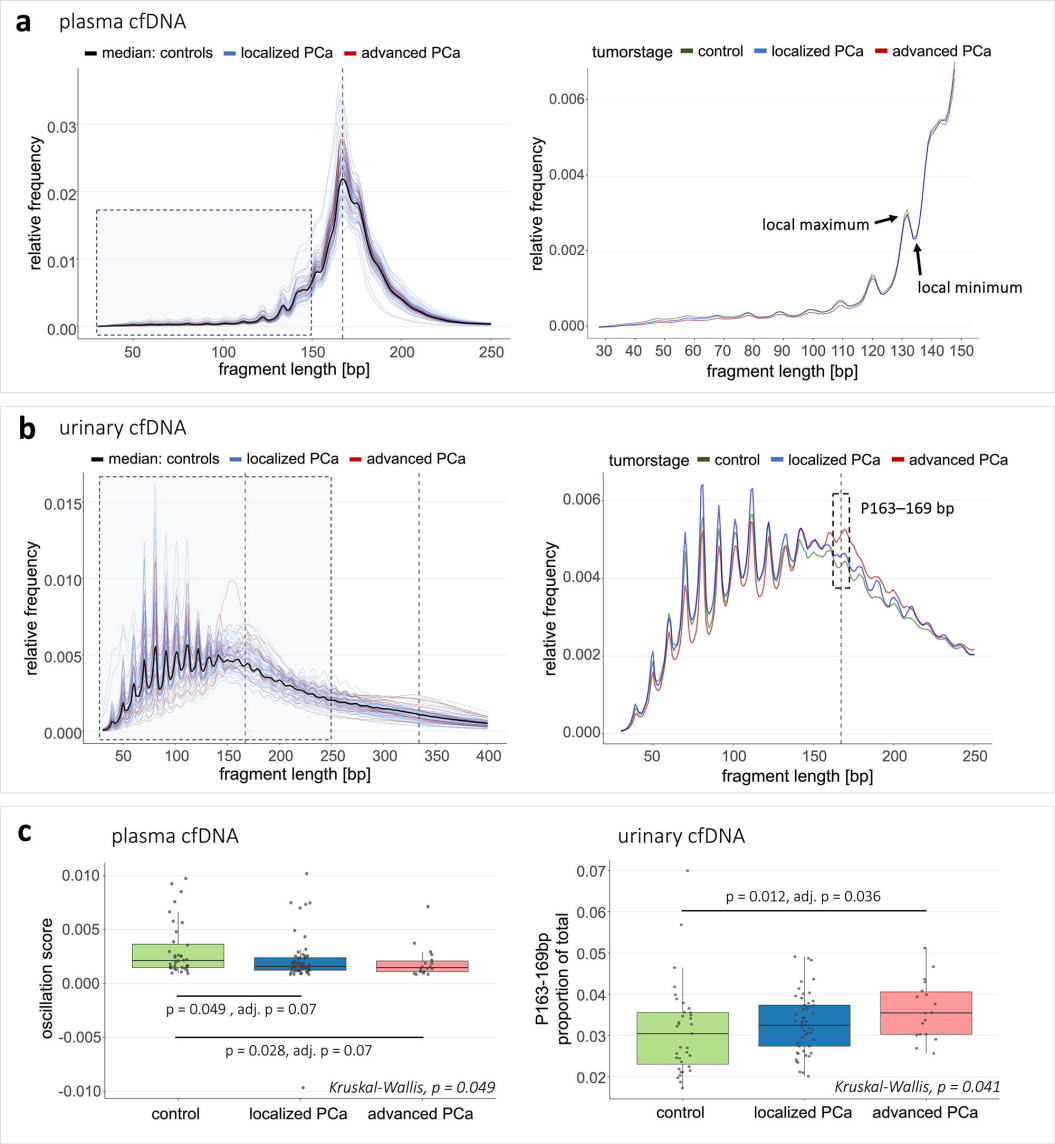
Fragmentation analysis of plasma and urinary cfDNA. **a** Left: Single plasma cfDNA fragmentation profiles of lPCa and aPCa patients, along with the median fragmentation profile of all cancer-free controls. Right: Plasma cfDNA fragmentation profiles (30–150 bp fragment length) exhibiting a 10 bp-oscillation pattern with local maxima and minima at 10 bp intervals. Median fragmentation profiles are shown for all samples from lPCa patients, aPCa patients, and cancer-free controls. **b** Left: Single urinary cfDNA fragmentation profiles of lPCa and aPCa patients, along with the median fragmentation profile of all cancer-free controls. Right: Urinary cfDNA fragmentation profiles, represented as median profiles of all samples from lPCa patients, aPCa patients, and cancer-free controls. Fragment length range 163–169 bp is highlighted. a, b: Y-axis: Relative frequencies of cfDNA fragments with specific length (bp) compared to all fragments (30–700 bp length). Vertical dotted gray line(s) indicate 167 bp and its multiples, 334 bp (2×167 bp). **c** Left: 10 bp-oscillation scores (calculated based on the deviation between the sum of the height of all local maxima and the sum of the depth of all local minima) in plasma cfDNA from cancer-free controls, lPCa patients, and aPCa patients. Right: P163–169 bp values in urinary cfDNA from cancer-free controls, lPCa patients, and aPCa patients. Left, right: Box plot center lines indicate the median, and boxes illustrate the interquartile range with Tukey whiskers. Each dot represents one sample. The three cohorts were compared using Kruskal-Wallis testing, followed by Dunn’s post hoc test. Only significant differences are shown. P163–169 bp = proportion of fragments with length 163-169 bp in relation to all fragments.

We analyzed global cfDNA fragmentation profiles, as well as various proportions and ratios of different fragment length ranges (Supplementary Fig. S7, S8) to identify tumor-specific patterns and infer ctDNA presence. Plasma and urinary cfDNA fragmentation showed distinct characteristics and differences between PCa patients and controls (Fig. 3a, b). Plasma cfDNA fragmentation profiles exhibited a prominent 167 bp peak, representing nucleosomal DNA wrapping (plus linker DNA), and additional peaks as multiples (Fig. 3a; Supplementary Fig. S9). Urinary cfDNA displayed a broader peak spanning 30–400 bp, though some samples contained an additional peak at ~167 bp, similar to plasma cfDNA (Fig. 3b; Supplementary Fig. S10). Both biofluids exhibited 10 bp-oscillation patterns in fragments <150 bp, characterized by periodic local maxima and minima, which is thought to arise from nucleosome positioning and periodic enzymatic cleavage of DNA^24,25^ (Fig. 3a, b). In urine, this pattern was more pronounced and extended to longer fragments (150–300 bp; Supplementary Fig. S10). Kolmogorov-Smirnov testing revealed significant differences in plasma cfDNA fragmentation between PCa and controls (*p* < 0.01), but not in urine due to higher intersample variability (Supplementary Fig. S9 + *S10*). The most tumor-informative plasma cfDNA fragmentation feature was the 10 bp-oscillation pattern, with scores progressively decreasing from controls to lPCa to aPCa (Fig. 3c). Twelve tumor samples harbored 10 bp-oscillation scores below the detectability threshold (5th percentile of controls), indicating ctDNA presence. In urine, PCa patients showed increased proportions of fragments with 163–169 bp length (Fig. 3c), especially for aPCa compared to controls (*p* = 0.012, adjusted *p* = 0.04), with one tumor sample exceeding the ctDNA detectability threshold.

### Multimodal analyses enhanced ctDNA detection in PCa

**Fig. 4:**
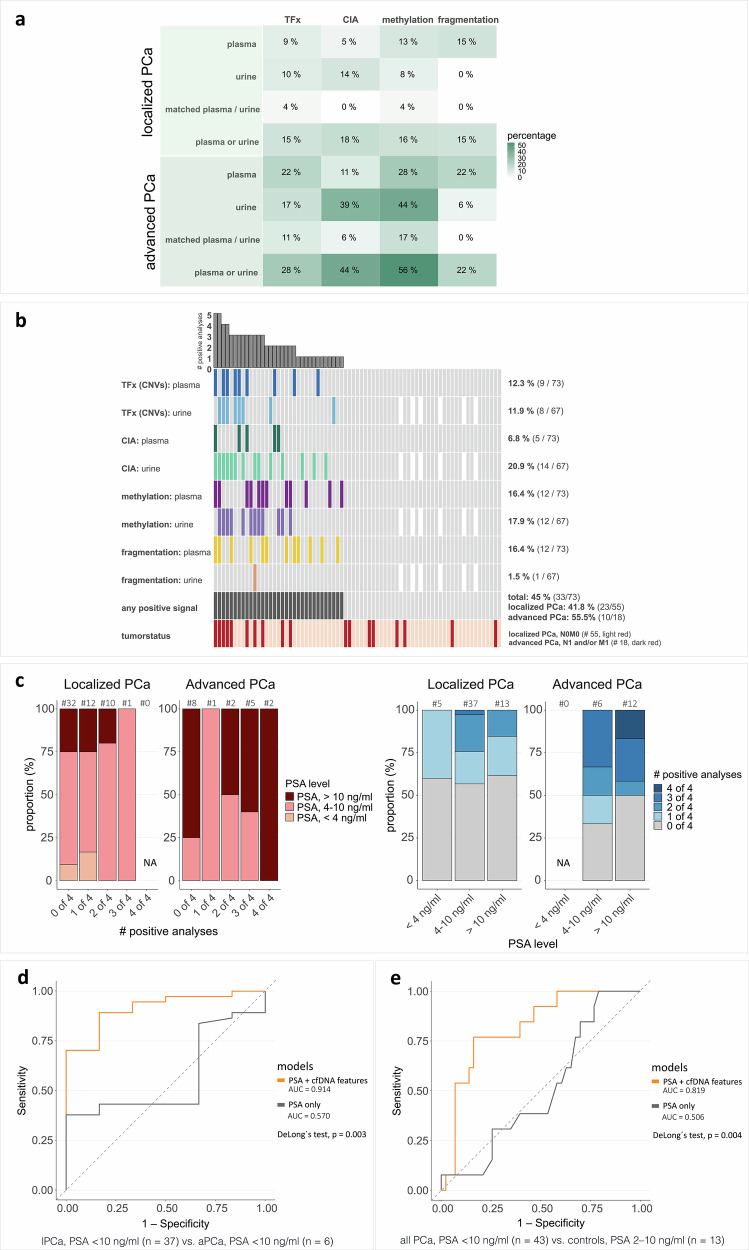
PCa detection and risk stratification based on multimodal LBx analyses. **a** Detection rates of tumor signals (ctDNA) in plasma and urine samples from lPCa (top) and aPCa (bottom) patients based on the four (epi)genomic analyses: 1) estimated TFx based on the CNV analysis with ichorCNA (plasma: analysis with in-silico size selection for 90–150 bp fragments, urine: analysis without size selection), 2) CIA score, 3) methylation score, 4) cfDNA fragmentation (10 bp-oscillation score in plasma cfDNA, P163–169 bp in urinary cfDNA). Detection rates (percentages, %) are shown separately for plasma and urine, for matched plasma and urine samples (ctDNA detected in both sample types from the same patient), and for the complementary analysis considering ctDNA detection in either plasma or urine. Overall, 68 PCa patients harbored matched plasma and urine samples; 4 lPCa patients with missing urine samples. **b** Oncoprint with results from genomic (estimated TFx based on CNVs, CIA score) and epigenomic (cfDNA fragmentation features, methylation score) analyses in plasma and urinary cfDNA. Patients with detectable ctDNA in the respective analyses are indicated with colored tiles. White tiles represent non-available urine samples (*n* = 4). The bar plot on top depicts the number of positive analyses per patient. The second-to-last row represents the number of PCa patients with positive signal in at least one analysis (“any positive signal”; colored tiles). **c** Association between the number of positive LBx analyses and PSA levels for lPCa and aPCa patients. Distribution of patients based on the number of positive tumor signals detected (0–4 out of 4 analyses) in relation to PSA levels (<4 ng/mL, 4–10 ng/mL, and > 10 ng/mL). Localized and aPCa are shown separately. Total numbers are depicted above each bar. Tumor signal positivity is determined using complementary analysis in plasma and urine across the four (epi)genomic assessments: TFx, CIA score, cfDNA fragmentation features, and methylation score. **d**, **e** ROC analysis of multimodal models combining PSA levels with cfDNA-derived features. ROC curves for GLMs based on PSA levels alone (PSA-only model) and PSA levels combined with cfDNA-derived features (PSA + cfDNA features model). **d** Models distinguishing lPCa from aPCa among patients with PSA < 10 ng/mL. **e** Models distinguishing PCa patients with PSA < 10 ng/mL from PSA-elevated controls who underwent PCa screening. AUC area under the curve, ctDNA circulating tumor-derived DNA, # number of, GLM generalized linear model, PSA prostate-specific antigen, ROC Receiver Operating Characteristic.

Single-parameter analyses yielded low to moderate ctDNA detection rates (DR), with plasma cfDNA fragmentation performing best in lPCa (DR = 15%), followed by the CIA score in urine (DR = 14%) and the methylation score in plasma (DR = 13%); other parameters showed detection rates ≤10% (Fig. 4a). In aPCa, detection rates increased across all features, with the methylation score (DR = 44%) and the CIA score (DR = 39%) in urine showing the best performance, followed by the methylation score (DR = 28%), TFx, and cfDNA fragmentation (both DR = 22%) in plasma (Fig. 4a). When evaluating the strongest cfDNA signal per patient across biofluids, by extracting the higher of the z-score–transformed plasma and urine values for TFx, CIA score, and methylation score, stage-dependent distributions were observed. In lPCa, the highest values for all three cfDNA features were more often detected in plasma, whereas in aPCa, they were more commonly detected in urine (Supplementary Fig. S11). Based on these highest per-patient cfDNA signals, aPCa patients exhibited significantly elevated results for TFx, CIA score, and methylation score compared with controls, and significantly higher CIA and methylation scores compared with lPCa patients (Supplementary Fig. S11). Complementary plasma-urine analysis improved ctDNA detection, yielding the highest detection rate based on the CIA score for lPCa (DR = 18%) and the methylation score for aPCa (DR = 56%).

Beyond complementarity of the two biofluids, combining genomic and epigenomic markers further enhanced ctDNA detection (Fig. 4b). In lPCa, ctDNA was detectable in 20% of plasma and 14% of urine samples based on one (epi)genomic feature; one urine sample was positive for three features, none for two or all four. In aPCa, 22% plasma samples were positive for one feature, 11% for two, and 6% for three or four features, respectively; 17% of urine samples were positive for one or three features, respectively. Considering all genomic and epigenomic cfDNA features in plasma and urine, ctDNA was detectable in 45% (33/73) of all PCa patients based on at least one positive result, including detection rates of 42% in lPCa and 56% in aPCa (Fig. 4b).

Among patients with aPCa, detectable ctDNA was found in four of nine individuals (44%) with lymph node–only disease (N1M0). Two were also classified as lymph node-positive on PET-CT, whereas three ctDNA-negative patients were lymph node-negative on imaging, but confirmed as positive following surgery. Among patients with distant metastases (N0/N1 M1), ctDNA was detected in six of nine cases (67%), including five with both nodal and distant involvement (Supplementary Table S3). Two of the three ctDNA-negative metastatic cases were N0M1 with bone-only metastases. CtDNA-positive M1 cases had a higher median PSA level (24.6 ng/mL) than ctDNA-negative cases (7.3 ng/mL). Regarding tumor characteristics, 80% of ctDNA-positive aPCa cases were locally advanced (T3–T4), and 67% harbored Gleason ≥8 (Supplementary Table S3). Only two M1 cases showed locally restricted tumors (T2a), and three were classified as Gleason 7a or 7b. In contrast, ctDNA-positive lPCa cases predominantly exhibited Gleason 7b or lower (15× Gleason 7a, 1× Gleason 7b, 1× Gleason 6), with only four tumors graded Gleason 8–9 (Supplementary Table S3). Most ctDNA-positive lPCa cases (74%) were locally restricted (T1–T2). Overall, ctDNA positivity was not significantly associated with Gleason grade or T stage in either localized or advanced disease.

Furthermore, ctDNA was present in 43% of lPCa and 67% of aPCa cases with PSA levels <10 ng/mL (Fig. 4c; Supplementary Tables S4, S5), including cases with PSA levels as low as 2.7 ng/mL. The median PSA level among ctDNA-positive patients was 8.1 ng/mL (range 2.7–249 ng/mL), with the highest values observed in ctDNA-positive M1 cases. Higher PSA levels correlated with an increasing number of positive analyses in both groups, especially in aPCa.

To evaluate whether cfDNA features provide additional discriminative value beyond PSA and T stage, we constructed generalized linear models (GLMs) combining clinical variables with ctDNA positivity. Adding binary ctDNA status (ctDNA positivity, yes/no) to the clinical model resulted in only marginal, non-significant increases in the area under the receiver operating characteristic (ROC) curve (AUC) for distinguishing aPCa from lPCa across the full cohort and among patients with PSA < 10 ng/mL. We next examined the utility of cfDNA analyses in an early screening context, where PSA levels are available prior to the availability of TNM staging information. Inclusion of quantitative cfDNA features (highest plasma or urine signal per patient for TFx, CIA score, methylation score (Supplementary Fig. S11); cfDNA fragmentation features: plasma 10 bp-oscillation score, urinary P163–169) together with PSA levels markedly improved discrimination between lPCa and aPCa among patients with PSA < 10 ng/mL. PSA alone yielded an AUC of 0.57 (95% confidence interval (CI) 0.35–0.79), whereas the combined model reached an AUC of 0.91 (95% CI 0.81–1.00; DeLong *p* = 0.0027; Fig. 4d). Bootstrap resampling confirmed this improvement (mean AUC 0.96; 95% CI 0.86–1.00) and the likelihood ratio test further indicated a significantly better model fit (χ² = 13.27, df = 7, *p* = 0.021).

A comparison between PCa cases with PSA 2–10 ng/mL and PSA-elevated controls provided additional evidence for these findings. PSA alone yielded an AUC of 0.51 (95% CI 0.34–0.68), whereas the multimodal cfDNA model significantly improved discrimination (AUC 0.82; 95% CI 0.70–0.94; DeLong *p* = 0.0037; Fig. 4e). Bootstrap resampling (mean AUC 0.85; 95% CI 0.74–0.97) and the likelihood ratio test (χ² = 12.72, df = 7, *p* = 0.026) supported these results.

## Discussion

This study evaluated genomic and epigenomic features in plasma and urinary cfDNA for ctDNA detection and risk stratification in newly diagnosed PCa. It presents a multimodal cfDNA analysis across two biofluids, offering a comprehensive approach not previously explored in primary PCa. Several key findings emerged from the assessment of multimodal cfDNA features, including genome-wide CNVs and chromosomal instability, methylation, and fragmentation patterns. First, genomic and epigenomic cfDNA features showed clear and statistically supported differences between PCa patients and cancer-free controls, with increasing tumor-associated signals observed in advanced disease, particularly evident in cfDNA methylation and chromosomal instability profiles. Second, the complementary assessment of matched plasma and urine samples improved ctDNA detectability and provided a more comprehensive tumor characterization than the analysis of either body fluid alone, underscoring the value of multi-source strategies. Third, combining genomic and epigenomic cfDNA features enhanced ctDNA detection compared to single-parameter analyses.

Integrating these findings, the multimodal analysis of genomic and epigenomic features in plasma and urinary cfDNA achieved an overall ctDNA detection rate of 45% in newly diagnosed PCa. CtDNA detectability was higher in aPCa, particularly in patients with distant metastases, and aPCa patients exhibited higher molecular burden as reflected by more frequent positive (epi)genomic cfDNA signals. Notably, ctDNA was also detectable in 43% of lPCa and 67% of aPCa cases with initial PSA levels <10 ng/mL, suggesting the potential of cfDNA analyses to complement PSA testing and refine risk stratification prior to prostate biopsy.

Among the assessed cfDNA features, epigenomic analyses revealed the best performance, with the 10 bp-oscillation score in plasma cfDNA excelling in lPCa, and the methylation score in both fluids in aPCa. The synoptic methylation score captured methylation levels of plasma and urinary cfDNA in PCa-informative, hypermethylated regions identified in PCa tissue and validated using an external dataset^20^. As regional hypermethylation accumulates with tumor progression^26,27^, methylation signals were increased in plasma and urine from aPCa patients in our cohort, and showed elevated and heterogeneous patterns in plasma from mCRPC patients in the external cohort by Chen et al.^21^. The authors also found a strong correlation between hypermethylated DMRs and ctDNA content in mCRPC plasma, though some mCRPC cases with low or undetectable ctDNA clustered closer to lPCa^21^. Urinary cfDNA from lPCa and aPCa patients in our cohort showed similar methylation patterns but generally higher levels than plasma, suggesting improved representation of PCa-specific methylation. This might reflect a higher ctDNA content in urine, possibly due to direct shedding from the urinary tract^28,29^, or other biological and technical differences between LBx sources. Furthermore, genome-wide plasma and urinary cfDNA methylation profiling identified hypermethylation in regions associated with *SOX2-OT*, *ZBTB46*, and *PTPRN2*, distinguishing mPCa from non-mPCa and controls. These genes were linked to tumorigenesis, androgen signaling, and metastasis^30–32^, reinforcing the value of cfDNA methylation analysis for PCa classification and risk stratification. Genomic cfDNA analyses contributed complementary information in plasma and urine for lPCa and aPCa, with increasing genomic instability observed in aPCa. Recurrent genomic alterations were present in both plasma and urine samples with increased TFx >10%, which aligned with known alterations in primary PCa^2,23^, supporting the potential for early genomic tumor characterization.

Our ctDNA detection rates aligned with prior studies, confirming higher sensitivities in mPCa^33^, while highlighting challenges in lPCa^13,14^. Genomic and epigenomic analyses of plasma and urinary cfDNA revealed both consistent and complementary patterns in ctDNA detectability, along with distinct fragmentation profiles. Characteristics of cfDNA fragmentation, previously described across tumor types^24,34,35^, result from differences in underlying biological processes such as cfDNA release mechanisms and nucleosomal packaging^36^. Urinary cfDNA is more heterogeneous in composition and less well understood than plasma cfDNA. One proposed mechanism involves glomerular filtration of cfDNA^28,36,37^, allowing for the selective passage of short plasma-derived fragments (100–250 bp), followed by enzymatic and mechanical degradation during transit through the urinary tract^38^. Longer cfDNA fragments ( > 1 kb) likely originate from urinary tract cells^39^, and ctDNA is directly shed from urological tumors. As a result, urinary cfDNA comprises a complex mixture of transrenal and locally released, tumor-derived and non-tumorous DNA. The presence of a strong tumor signal in urinary cfDNA, likely due to direct ctDNA shedding, was supported by selected clinical cases in our cohort. In some patients with bladder-invading tumors, urinary cfDNA displayed a prominent 160–180 bp peak, similar to plasma cfDNA profiles, and showed detectable ctDNA based on genomic or combined genomic and epigenomic analyses.

While complementary plasma-urine analyses are rare in PCa^40,41^, multimodal strategies have reinforced their potential for improved diagnostic accuracy in other malignancies^22,42–44^. Our analysis incorporated genomic and epigenomic features previously demonstrated to contribute to tumor characterization. Methylation analyses showed promise for sensitive ctDNA detection through targeted approaches in plasma and urine from PCa patients^45,46^, as well as genome-wide methylation profiling coupled with machine-learning classifiers, which distinguished mPCa from lPCa or controls^21,47^. Mouliere et al. observed that cfDNA fragmentation patterns, including the 10 bp-oscillation score, differed between tumor and control samples, indicating their relevance as biomarkers in tumor characterization^18,48^. The authors observed reduced 10 bp-oscillation amplitudes in cerebrospinal fluid from glioma patients with detectable CNVs compared to CNV-negative patients^49^. Similarly, plasma cfDNA oscillation scores progressively declined from controls to lPCa and further to aPCa in our cohort. CNV analyses in plasma cfDNA using lcWGS were shown to detect ctDNA, predominantly in mPCa^14,50^. However, ctDNA detection in plasma from lPCa remains challenging, despite the application of combined genomic approaches such as lcWGS for CNV analysis and targeted sequencing for mutation detection, as reported by Hennigan et al.^14^. Most lPCa patients in this study harbored PSA levels below 10 ng/mL, and even the patient with the highest level (43.6 ng/mL) showed no detectable CNVs^14^. Within our cohort, 70% of PCa patients with detectable ctDNA in plasma or urine, and all with detectable ctDNA in urine, also had PSA levels below 10 ng/ml, underscoring the value of integrated genomic and epigenomic analyses.

Multimodal LBx has the potential to complement current diagnostics. Our findings indicated that higher PSA levels correlated with increased ctDNA detection, particularly in aPCa. For PSA > 10 ng/mL, positive LBx findings could help distinguish aggressive lPCa from aPCa, guiding risk-stratified treatment decisions toward surgery, multimodal therapy, or systemic treatment. CtDNA was detectable in cases with PSA < 10 ng/mL, emphasizing its promising diagnostic value for patients with inconclusive PSA values, refining risk stratification at multiple clinical decision points. CfDNA analyses may support decision-making in cases of ambiguous imaging findings and guide further diagnostics (i.e., prostate biopsy), potentially reducing unnecessary procedures. To further explore this, we examined the potential value of cfDNA analyses in early PCa assessment, where TNM staging information is not yet available, and clinical decision-making relies primarily on PSA testing. In cases with intermediate PSA levels (2–10 ng/mL), GLMs developed to predict lPCa versus aPCa or tumor versus non-tumor status demonstrated a significant improvement in discriminatory performance when multimodal cfDNA features were incorporated alongside PSA levels compared with PSA alone. These findings suggest that incorporating cfDNA-derived parameters into a multimodal model may provide added clinical value during early diagnostic decision-making in patients with inconclusive PSA levels. However, the modest sample sizes and intercorrelations among cfDNA features warrant cautious interpretation. Model overfitting cannot be excluded and may partially account for the observed performance gains. Because of the limited cohort size, no independent training and test set validation could be performed. Optimization and external validation in larger, independent cohorts will be necessary to strengthen these findings.

Future research benefits from technological and bioinformatic advances to improve diagnostic sensitivity, particularly in early-stage PCa. Integrating multiple cfDNA parameters, as demonstrated in this study, or combining them with further data modalities (e.g., imaging) represent promising strategy for improved risk stratification, guiding clinical decision-making. CfDNA monitoring could refine therapeutic decisions at initial diagnosis and provide insights into treatment response and tumor progression for patients under systemic therapy. Overall, our findings can be viewed as a proof-of-concept, highlighting the potential of multimodal cfDNA profiling, but requiring further refinement and validation in larger prospective studies to assess its clinical applicability^51^^,52^.

Limitations of this study include the relatively small sample size, which constrained statistical power and limited the generalizability of our findings, necessitating validation and refinement of detection thresholds in larger, independent cohorts. Although our multimodal approach improved ctDNA detection compared to single-parameter assays, overall sensitivities remained low, particularly in lPCa, underscoring the challenges of early-stage PCa detection. CtDNA was not detectable in all aPCa patients, which may reflect both biological and technical factors, e.g., interpatient variability in ctDNA shedding, differences in metastatic burden or disease biology, as well as limitations in current assay sensitivity. Furthermore, cfMeDIP-seq enabled cost-effective, practically applicable genome-wide methylation profiling and demonstrated potential for methylation biomarker assessment, but also harbored limitations, including antibody-dependent enrichment biases, restricted base-level resolution, and underrepresentation of hypomethylated regions^53^.

In conclusion, this proof-of concept study indicates that the integration of multiple genomic and epigenomic cfDNA biomarkers from two LBx sources can improve molecular characterization and ctDNA detection in newly diagnosed PCa, with potential to support future risk stratification and inform clinical decision-making. Multimodal LBx analyses may ultimately complement PSA testing, particularly in cases with inconclusive or low PSA levels, thereby contributing to more refined risk assessment at diagnosis. The moderate costs and rapid turnaround time of cfDNA-based assays support their feasibility for clinical implementation. With ongoing technological progress, multimodal LBx approaches offer potential to improve clinical decision-making and contribute to future advancements in precision oncology, with possible translation to other urological malignancies.

## Methods

### Study patients

Seventy-three PCa patients at initial diagnosis and 36 cancer-free controls were recruited at Heidelberg University Hospital (June 2021–November 2022). PCa patients were enrolled at the time of initial diagnosis without prior treatment. The control cohort included men who either underwent PCa screening with no signs of malignancy on prostate biopsy or were treated for benign urological conditions. Blood and urine samples were collected prior to examinations, i.e., prostate biopsy or surgery. Comprehensive clinical data were available for all men. PSA levels were obtained from internal measurements or external documentation. The study was approved by the ethical committee of the University of Heidelberg (Approval No. S-130/2021) and was performed in accordance with the Declaration of Helsinki. Written informed consent was obtained from all participants prior to study inclusion.

### Sample preparation and sequencing

Peripheral blood and urine were processed by double-spin centrifugation within a maximum of 6 hours. CfDNA was isolated from 1–5.5 mL of plasma and 9.5–19 mL of urine supernatant, respectively, using the QIAamp MinElute ccfDNA Kit (Qiagen). Eight fresh-frozen PCa tissue samples were provided by the Tissue Bank of the National Center of Tumor Diseases (NCT), Heidelberg. Genomic DNA (gDNA) from PCa tissue and matched buffy coat was extracted using the AllPrep DNA/RNA/Protein Mini Kit (Qiagen) and QIAamp DNA Mini and Blood Mini Kit (Qiagen), respectively, followed by shearing with the M220 Focused Ultrasonicator (Covaris) to generate uniform DNA fragments of ~150–220 bp length. Libraries were prepared from 2.4–7 ng cfDNA or 100 ng sheared gDNA, using the KAPA HyperPrep Kit (Roche) with NEBNext UDI-UMI Adaptors (New England Biolabs). Eighty percent of the volume of each library was processed using the (cell-free) methylated DNA immunoprecipitation workflow, as described by Shen et al.^53^, with minor adaptations. The remaining 20% of the library volume was retained without enrichment, serving as a reference control for methylation enrichment quality assessment and as input for lcWGS. Finally, enriched and non-enriched libraries were amplified for 12–13 (gDNA: eight) and 8–9 (gDNA: six) cycles of polymerase chain reaction, respectively, pooled equimolarly, and paired-end sequenced (2× 100 bp) on the NovaSeq 6000 platform (Illumina).

### Sequencing data processing

Raw sequencing data from lcWGS (non-enriched libraries) and (cf)MeDIP-seq (enriched libraries) were processed with a custom pipeline, including adapter trimming, read alignment to the human reference genome (hg19), deduplication based on the information from unique molecular identifiers (UMI), quality filtering, and final quality control. Data processing was performed using an in-house Nextflow pipeline^54^, and workflow components were sourced from nf-core^55^, with additional custom modules and sub-workflows.

### Genome-wide methylation profiling

Genome-wide methylation profiling of LBx and tissue samples was performed based on the (cf)MeDIP-seq data, using the R package MESA v0.2.2^56^. Relative methylation signals, expressed as normalized reads per kilobase million (nrpkm), were assessed in genome-wide 300 bp windows and were transformed to absolute methylation levels (β-values), scaled from 0 (unmethylated) to 1 (fully methylated). DMRs were determined in plasma and urine based on significant differences (*p* < 0.05) in absolute methylation levels between PCa patients and controls. Methylation differences were expressed as logFC, with positive and negative logFC indicating hyper- and hypomethylation relative to the reference group, respectively. DMRs were annotated for genomic regions and CpG-associated landscapes using an annotation function included in the R package MESA v0.2.2^56^ (based on the ChIPseeker R package^57^). Common DMRs between groups or between plasma and urine samples, respectively, were identified with ChIPpeakAnno v.3.24.2^58^, and visualized in Venn diagrams.

DMRs were also determined in eight PCa tissue samples relative to matched buffy coat. Significant, hypermethylated DMRs (adjusted *p* < 0.01, logFC > 2) in PCa tissue were compared to the top 100 hypermethylated DMRs (500 bp windows, ranked by the lowest *p* values) from an external MeDIP-seq dataset of 51 primary PCa and 53 normal prostate tissue samples, published by Börno et al. (Supplementary Data, Table S3a)^20^. The overlapping 300 bp regions from this comparison were selected as methylation markers for LBx analysis. A methylation score was calculated for each plasma and urine sample as the median β-value across the selected regions, serving as a synoptic measure of the methylation status in PCa patients and controls. Additionally, methylation markers were assessed in an external cfMeDIP-seq dataset from Chen et al.^21^. Thereby, hierarchical clustering (R package pheatmap v.1.0.12^59^) and PCA (R package stats v.4.0.0^60^) were performed on absolute cfDNA methylation levels in the selected biomarker regions, derived from 133 plasma samples from lPCa (*n* = 30) and mCRPC (*n* = 103) patients.

### Copy number profiling and chromosomal instability analysis

Genome-wide CNV profiling of plasma and urinary cfDNA was performed based on lcWGS data using the ichorCNA algorithm^61^. Briefly, genomes were segmented into 1000-kilobase (kb) bins, read counts were computed, and normalized for GC content and mappability. Copy number states were inferred by comparison to a panel of normals (PoN) used as a copy-neutral reference. For tumor samples, a PoN was generated by aggregating all control samples; for each control sample, an individual PoN was created by excluding the respective sample from the tumor PoN. CNV segmentation was performed using a hidden Markov model (HMM), and TFx were estimated from predicted large-scale CNVs. This analysis was conducted on all sequencing reads (30–700 bp), followed by an additional in-silico size selection. For plasma cfDNA, size selection was applied to the 90–150 bp fragment length range, previously shown to enhance ctDNA detection in the CNV analysis^18,22^. For urinary cfDNA, different size ranges were tested to determine the most effective range for improving CNV detection (see Supplementary Fig. S4). CNV profiling was also performed on the lcWGS data from eight PCa tissues and matched buffy coat samples, with buffy coat data serving as a copy-neutral reference (PoN) for each tissue sample. In addition to the CNV analysis, genomic instability of plasma and urinary cfDNA was assessed, and a synoptic CIA score was calculated for each sample, following previously published approaches^62,63^ with minor adaptations.

### Plasma and urinary cfDNA fragmentation analysis

Plasma and urinary cfDNA fragment distributions were analyzed by assessing insert sizes (restricted to 30–700 bp lengths) from lcWGS data. Relative frequencies were calculated as the proportion of each size within this range, and cumulative frequencies were also derived. Fragmentation analysis was adapted from Mouliere et al.^18,48,49^. Proportions of predefined fragment length ranges, as well as ratios of short-to-long fragments and nucleosome-associated ranges were computed, and statistically compared between tumor and control samples. Additionally, the 10 bp-oscillation pattern in plasma and urinary cfDNA was examined in 30–150 bp and 150–300 bp fragment length ranges.

### Detection of circulating tumor-derived DNA (ctDNA) in plasma and urine

CtDNA detection in plasma and urine from PCa patients was assessed using the estimated TFx from the CNV analysis, the CIA score, the methylation score, and two cfDNA fragmentation features. TFx, CIA score, and methylation score were evaluated in both plasma and urinary cfDNA, whereas two different fragmentation features were used for plasma (10 bp-oscillation score) and urine (P163–169bp). TFx, CIA score, methylation score, and P163–169 bp in urinary cfDNA used a ctDNA detectability threshold set at the 95th percentile of control samples; tumor samples with values above this threshold were considered positive. The ctDNA detectability threshold for the 10 bp-oscillation score in plasma cfDNA (30–150 bp fragment length) was set at the 5th percentile of controls, classifying tumor samples with a lower value as positive.

### Statistical analyses and data visualization

Statistical analyses were conducted in R v4.0.0^60^ and statistical significance was assessed using the Kruskal-Wallis test with Dunn’s post hoc test, Wilcoxon rank-sum test, Fisher’s exact test, and Spearman’s correlation. Cumulative frequency distributions of cfDNA fragmentation were compared with Kolmogorov-Smirnov testing. *P* values were always adjusted for multiple testing with Benjamini-Hochberg’s method. Significance was set at (adjusted) *p* < 0.05, unless stated otherwise. The ctDNA detectability threshold for all evaluated (epi)genomic markers was defined as >95th percentile of the control cohort, with the exception of the 10 bp-oscillation score (<5th percentile). Plots were generated in R v4.0.0^60^ using the R package ggplot2 v.3.4.2^64^. The R packages plotly v.4.10.2^65^ and ggfortify v.0.4.17^66^ were used for PCA plots, displaying the first two principal components with their explained variance on the x- and y-axes, respectively.

GLMs were constructed to distinguish lPCa from aPCa and to differentiate PCa patients from PSA-elevated controls. Different model types were assessed: models including PSA and clinical T stage or PSA alone together with ctDNA positivity, and models integrating PSA levels with quantitative cfDNA features. All continuous variables were z-score transformed prior to model fitting. Model performance was evaluated using the AUC of the ROC curve. Differences between AUCs were assessed using DeLong’s test, and model fit was evaluated using likelihood ratio tests. Robustness of AUC estimates was further examined through bootstrap resampling (500 iterations). GLMs were constructed using base R functions, and AUC values, ROC curves, and DeLong tests were generated using the R package pROC v1.18.2^67^.

### Usage of generative Artificial Intelligence (AI) and AI-assisted technologies

During the preparation of this work, the authors used ChatGPT-4o in order to improve language and readability. After using this tool, the authors reviewed and edited the content as needed, and took full responsibility for the content of the publication.

## Supplementary information

Supplementary information

## Data Availability

The data sets generated and analyzed during the current study are available from the corresponding author on reasonable request. LcWGS data and (cf)MeDIP-seq data that support the findings of this study are deposited in the European Genome-Phenome Archive (EGA) under accession number EGAS00001008195. Sequencing data from Chen et al. (10.1038/s41467-022-34012-2) was previously deposited to the EGA under accession numbers EGAD00001007972, EGAD00001008711, EGAD00001008712, and EGAD00001008713.

## Code availability

All R packages and bioinformatic tools used in this study are publicly available, as described in the Methods section. Any additional information, including custom code for data processing and visualization, required to reanalyze the data reported in this work, is available from the corresponding author upon reasonable request.
