# Supplementary material for: Multimodal plasma and urinary cell-free DNA profiling improves risk stratification in newly diagnosed prostate cancer
Source: NPJ Precis Oncol. 2026 Mar 2;10:115. doi: 10.1038/s41698-026-01343-y (PMC12996294; doi:10.1038/s41698-026-01343-y)
Supplement: Supplementary file 1 — Supplementary information [file 41698_2026_1343_MOESM1_ESM.pdf]

Supplementary Material

Multimodal plasma and urinary cell-free DNA profiling improves risk stratification in newly diagnosed prostate cancer

Anja Lisa Riediger, Samaneh Eickelschulte, Florian Janke, Daniela Janscho, Olga Lazareva, Daniel Hübschmann, Stefan Duensing, Oliver Stegle, Holger Sültmann and Magdalena Görtz

Supplementary Tables

Table S1: Overview of raw and final quality-filtered sequencing reads, as well as CpG-site coverage metrics from lcWGS and (cf)MeDIP-seq data
Table S2: Overview of detectable DMRs between PCa patients and controls in plasma and urinary cfDNA
Table S3: Clinical characteristics of PCa patients with positive ctDNA signals based on multimodal LBx analyses
Table S4: Overview of PCa patients with positive signals in genomic and epigenomic LBx analyses by PSA range and PCa stage
Table S5: Distribution of positive signals across genomic and epigenomic LBx analyses by PSA range and PCa stage

Supplementary Figures

Figure S1: Genome-wide methylation profiling in plasma and urinary cfDNA
Figure S2: Genome-wide methylation profiling in genomic DNA from PCa tissue
Figure S3: Clustering based on β-values in 67 marker regions for plasma samples from lPCa or mCRPC patients from an external cohort
Figure S4: Genome-wide assessment of CNVs in plasma and urinary cfDNA
Figure S5: Correlation between genomic biomarkers in plasma and urinary cfDNA
Figure S6: Genome-wide assessment of CNVs in genomic DNA from fresh-frozen PCa tissue samples
Figure S7: Plasma cfDNA fragmentation analysis
Figure S8: Urinary cfDNA fragmentation analysis
Figure S9: Relative and cumulative frequency distributions of plasma cfDNA fragmentation
Figure S10: Relative and cumulative frequency distributions of urinary cfDNA fragmentation
Figure S11: Comparison of highest per-patient cfDNA feature values across controls, lPCa, and aPCa patients

Supplementary Tables

Supplementary Table S1: Overview of raw and final quality-filtered sequencing reads, as well as CpG-site coverage metrics from lcWGS and (cf)MeDIP-seq data.

	**total raw reads,** M	**total final reads,** M	**final reads,** %	**genome-wide CpGs covered,** %	**covered CpGs with ≥ 5 reads,** %
cfDNA, lcWGS	91.0 [45.4 ; 141.2]	72.4 [35.5 ; 109.8]	80.28 [70.16 ; 84.56]	88.9 [67.7–95.8]	5.8 [0.3–32.4]
plasma, lcWGS	88.2 [56.3 ; 117.0	71.2 [45.3 ; 95.2]	81.32 [72.54 ; 84.56]	89.5 [75.6–94.9]	6.7 [0.8–22.5]
urine, lcWGS	92.3 [45.4 ; 141.2]	73.2 [35.5 ; 109.8]	79.51 [70.16 ; 83.36]	88.4 [67.7–95.8]	5.7 [0.3–32.4]
cfDNA, cfMeDIP	90.1 [74,626 ; 218.9]	60.2 [53,358 ; 137.2]	66.78 [38.55 ; 76.01]	79.1 [54.4–93.1]	36.0 [11.2–65.1]
plasma, cfMeDIP	91.6 [70.2 ; 200.5]	62.4 [36.2 ; 136.4]	68.24 [50.82 ; 76.01]	78.6 [54.4–88.5}	37.2 [13.8–57.7]
urine, cfMeDIP	88.4 [74,626 ; 218.9]	56.2 [53,358 ; 137.2]	64.04 [38.55 ; 75.45]	80.1% [61.1–93.1]	34.9 [11.2–65.1]
genomic DNA, lcWGS	76.6 [68.2 ; 87.9]	62.4 [55.8 ; 71.2]	81.76 [80.78 ; 82.59]	87.2 [82.8–89.1]	3.6 [2.3–5.1]
tissue, lcWGS	78.4 [68.2 ; 83.7]	63.5 [55.8 ; 68.0]	81.43 [80.78 ; 82.01]	86.0 [82.8–89.1]	3.0 [2.3–5.1]
buffy coat, lcWGS	73.3 [70.1 ; 87.9]	60.1 [57.9 ; 71.2]	82.07 [81 ; 82.59]	87.8 [86.2–89.1]	4.0 [3.0–5.1]
genomic DNA, MeDIP	74.4 [49.7 ; 108.0]	54.1 [33.9 ; 80.0]	72.38 [67.44 ; 74.73]	76.1 [69.9–81.0]	35.9 [24.7–46.9]
tissue, MeDIP	73.3 [49.7 ; 80.2]	52.6 [33.9 ; 57.7]	70.58 [67.44 ; 74.73]	75.5 [69.9–77.4]	34.7 [24.7–37.7]
buffy coat, MeDIP	74.7 [68.3 ; 108.0]	54.3 [50.3 ; 80.0]	73.02 [72.07 ; 74.02]	76.8 [74.2–81.0]	37.0 [34.7–46.9]

CpG-site coverage metrics represent (1) the proportion of all genomic CpG sites covered by at least one sequencing read, and (2) the proportion of captured CpG sites covered by more than five reads. Abbreviations: cfDNA = cell-free DNA, (cf)MeDIP-seq = (cell-free) methylated DNA immunoprecipitation sequencing, lcWGS = low-coverage whole-genome sequencing, M = Million

Supplementary Table S2: Overview of detectable DMRs between PCa patients and controls in plasma and urinary cfDNA.

**comparison**	**# samples**<br>total<br>(cohort 1 vs. cohort 2)	**PLASMA**<br># significant windows (DMRs)	**URINE**<br># significant windows (DMRs)
**PCa (all)** vs. **all controls**	plasma: 109 (73 vs. 36)<br>urine: 102 (67 vs. 35)	0 / 1,168,518<br>windows	0 / 1,195,872<br>windows
**aPCa (M1)** vs. **all controls**	plasma: 45 (9 vs. 36)<br>urine: 44 (9 vs. 35)	712 / 980,927<br>windows<br>*hyper: 615*<br>*hypo.: 97*	48 / 992,774<br>windows<br>*hyper: 29*<br>*hypo.: 19*
**aPCa (all)** vs. **all controls**	plasma: 54 (18 vs. 36)<br>urine: 53 (18 vs. 35)	0 / 1,008,208<br>windows	2 / 1,030,695<br>windows
**lPCa high-risk** vs. **all controls**	plasma: 45 (9 vs. 36)<br>urine: 44 (9 vs. 35)	0 / 945,422<br>windows	0 / 980,210<br>windows
**lPCa intermediate-risk** vs. **all controls**	plasma: 78 (42 vs. 36)<br>urine: 72 (37 vs. 35)	0 / 1,112,508<br>windows	0 / 1,127,546<br>windows
**lPCa (all)** vs. **all controls**	plasma: 91 (55 vs. 36)<br>urine: 84 (49 vs. 35)	0 / 1,134,888<br>windows	0 / 1,152,290<br>windows
**lPCa (all) + aPCa (N1 M0)** vs. **all controls**	plasma: 100 (64 vs. 36)<br>urine: 93 (58 vs. 35)	0 / 1,147,368<br>windows	0 / 1,171,278<br>windows
**aPCa (M1)** vs. **aPCa (N1 M0)**	plasma: 18 (9 vs. 9)<br>urine: 18 (9 vs. 9)	2 / 838,576<br>windows	2 / 821,533<br>windows
**aPCa (all)** vs. **lPCa (all)**	plasma: 73 (18 vs. 55)<br>urine: 67 (18 vs. 49)	0 / 1,115,932<br>windows	0 / 1,112,691<br>windows
**aPCa (M1)** vs. **lPCa (all) + aPCa (N1 M0)**	plasma: 73 (9 vs. 64)<br>urine: 67 (9 vs. 58)	890 / 1,115,932<br>windows<br>*hyper: 835*<br>*hypo.: 55*	64 / 1,112,691<br>windows<br>*hyper: 44*<br>*hypo.: 20*
**aPCa (M1)** vs. **lPCa intermediate-risk**	plasma: 51 (9 vs. 42)<br>urine: 46 (9 vs. 37)	309 / 1,064,258<br>windows	18 / 1,048,296<br>windows
**aPCa (all)** vs. **lPCa intermediate-risk**	plasma: 60 (18 vs. 37)<br>urine: 55 (18 vs. 37)	0 / 1,086,590<br>windows	0 / 1,081,990<br>windows
**aPCa (M1)** vs. **lPCa high-risk**	plasma: 18 (9 vs. 9)<br>urine: 18 (9 vs. 9)	29 / 830,239<br>windows	0 / 806,021<br>windows
**aPCa (all)** vs. **lPCa high-risk**	plasma: 27 (18 vs. 9)<br>urine: 27 (18 vs. 9)	0 / 895,667<br>windows	0 / 889,649<br>windows

DMR analysis based on β-values in 300 bp windows, including only windows in which at least one sample harbors >2 NRPKMs, multiple testing with Benjamini-Hochberg (adjusted p value < 0.05). Abbreviations: DMR = differentially methylated region, hyper = hypermethylated, hypo = hypomethylated, M0/M1 = absence/presence of distant metastases, N0/N1 = absence/presence of lymph node metastases, NRPKM = normalized reads per kilobase million mapped reads, # = number of, PCa = prostate cancer (aPCa = advanced PCa, lPCa = localized PCa)

Supplementary Table S3: Clinical characteristics of PCa patients with positive ctDNA signals based on multimodal LBx analyses.

TNM stage	Gleason score	PSA level (ng/ml)
ctDNA-positive, localized PCa patients		
cT1c cN0 cM0	7a	11.6
cT1c cN0 cM0	8	9.6
cT1c cN0 cM0	7b	8.6
cT1c cN0 cM0	7a	5.5
cT1c cN0 cM0	7a	8.1
cT1c cN0 cM0	6	9.1
cT2c cN0 cM0	7b	11.0
pT2a pN0 (0/16) cM0	7a	2.7
pT2c pN0 (0/13) cM0	7b	7.4
pT2c cN0 cM0	7a	3.2
pT2c cN0 cM0	7a	4.8
pT2c cN0 cM0	7a	5.6
pT2c cN0 cM0	7a	6.2
pT2c cN0 cM0	7a	6.3
pT2c cN0 cM0	7a	8.1
pT2c cN0 cM0	7a	8.93
pT2c cN0 cM0	7a	11.5
pT3a pN0 (0/22) cM0	9	21.4
pT3a pN0 (0/19) cM0	7a	4.3
pT3a cN0 cM0	7a	5.1
pT3a cN0 cM0	7a	7.7
pT3b pN0 (0/22) cM0	9	9.5
pT3b pN0 (0/14) cM0	9	26.4
ctDNA-positive, advanced PCa patients (N1 M0)		
pT3a pN1 (1/12) cM0	7b	4.1
pT3b pN1 (1/23) cM0	9	7.5
pT3b pN1 (2/22) cM0	9	19.3
cT4 cN1 cM0	8	4.3
ctDNA-positive, advanced PCa patients (N0/N1 M1)		
cT2a cN0 cM1	9	90.0
cT2a cN1 cM1b	7b	18.3
pT3b pN1 (2/21) cM1b	7a	11
cT4 cN1 cM1	10	7.1
cT4 cN1 cM1b	8	30.9
cT4 cN1 cM1	8	249.0

Abbreviations: cM = clinical assessment of distant metastases, LBx = Liquid Biopsy, cN/pN = clinical/pathological assessment of lymph node metastases: for pN, the number of positive lymph nodes among all examined nodes is provided in brackets, PSA = prostate-specific antigen, cT/pT = clinical/pathological assessment of the primary tumor’s extent

Supplementary Table S4: Overview of PCa patients with positive signals in genomic and epigenomic LBx analyses by PSA range and PCa stage.

		localized PCa		advanced PCa	
**analysis type**	**PSA range**	**# patients**	**PSA levels** median, range	**# patients**	**PSA levels** median, range
TFx	< 4 ng/ml	0	x	0	x
	4-10 ng/ml	7	7.7 [4.3 - 8.1]	2	4.3 ; 7.25
	> 10 ng/ml	1	11	3	30.9 [11 - 90]
CIA score	< 4 ng/ml	1	2.7	0	
	4-10 ng/ml	7	8.1 [4.3 - 9.6]	3	7.1 [4.3 - 7.5]
	> 10 ng/ml	2	11 ; 26.4	5	30.9 [11 - 249]
Methylation score	< 4 ng/ml	0	x	0	x
	4-10 ng/ml	7	8.1 [6.2 - 9.6]	4	5.7 [4.1 - 7.5]
	> 10 ng/ml	2	11.6 ; 21.4	6	25.1 [11 - 249]
cfDNA fragmentation	< 4 ng/ml	1	3.2	0	x
	4-10 ng/ml	6	7.7 [4.8 - 9.5]	0	x
	> 10 ng/ml	2	11.5 ; 11.6	5	19.3 [11 - 249]

Number of lPCa and aPCa patients (stratified by PSA range) with detectable ctDNA in plasma and/or urine in the four analysis types. The median PSA level and range for each subgroup are provided (median [range]). For groups with fewer than three patients, single values were reported instead of the median. Abbreviations: CIA = chromosomal instability analysis, TFx = tumor fraction

Supplementary Table S5: Distribution of positive signals across genomic and epigenomic LBx analyses by PSA range and PCa stage.

		localized PCa		advanced PCa	
**# positive analyses**	**PSA range**	**# patients**	**PSA levels** median, range	**# patients**	**PSA levels** median, range
**0 out of 4**	< 4 ng/ml	3	3 [2.7 - 3.9]	0	x
	4-10 ng/ml	21	6.8 [4.1 - 9.6]	2	7.2, 7.3
	> 10 ng/ml	8	19.56 [12.1 - 40]	6	22 [17 - 33]
**1 out of 4**	< 4 ng/ml	2	2.7 ; 3.2	0	x
	4-10 ng/ml	7	6.3 [4.8 - 9.1]	1	4.1
	> 10 ng/ml	3	21.4 [11.5 - 21.4]	0	x
**2 out of 4**	< 4 ng/ml	0	x	0	x
	4-10 ng/ml	8	7.9 [4.3 - 9.6]	1	7.1
	> 10 ng/ml	2	11.0 ; 11.6	1	18.3
**3 out of 4**	< 4 ng/ml	0	x	0	x
	4-10 ng/ml	1	8.1	2	4.3 ; 7.5
	> 10 ng/ml	0	x	3	90 [19.3 - 249]
**4 out of 4**	< 4 ng/ml	0	x	0	x
	4-10 ng/ml	0	x	0	x
	> 10 ng/ml	0	x	2	11 ; 30.9

Number of positive lPCa and aPCa patients (stratified by PSA range), according to the number of analyses (0–4 out of 4) with detectable ctDNA in plasma and/or urine. For each PSA category, the number of lPCa and aPCa patients with positive signals is reported, along with the median PSA level and range for each group (median [range]). For groups with fewer than three patients, single values were reported instead of the median.

Supplementary Figures

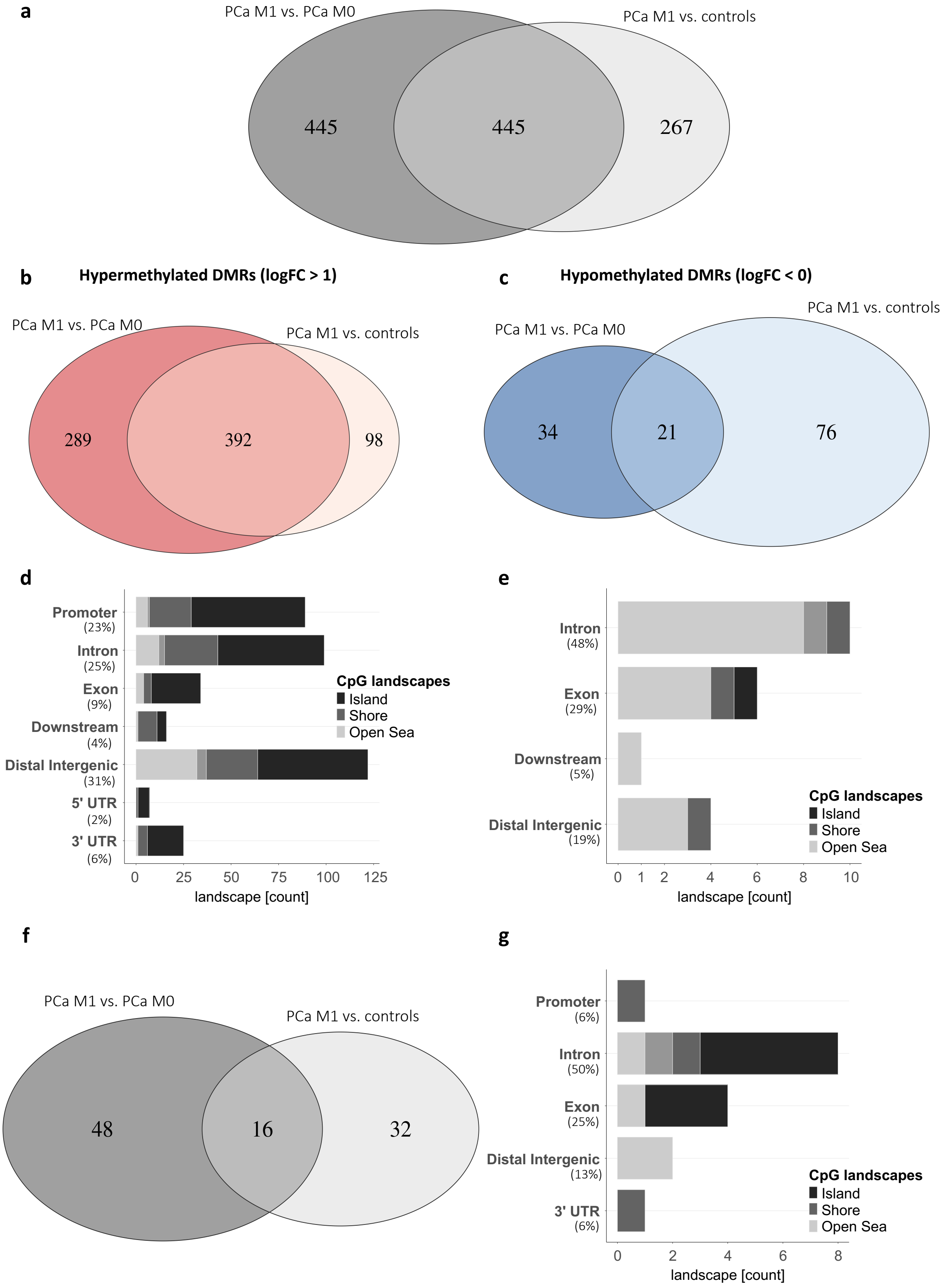

Supplementary Figure S1: Genome-wide methylation profiling in plasma and urinary. **a)** Common significant DMRs in plasma cfDNA between the comparisons metastatic PCa patients (PCa M1) vs. controls or PCa M1 vs.

PCa patients without distant metastases (PCa M0). **b)** Common significant, hypermethylated DMRs (logFC > 1) in plasma cfDNA between the comparisons PCa M1 vs. controls or PCa M1 vs. PCa M0. **c)** Common significant, hypomethylated DMRs (logFC < 0) in plasma cfDNA between the comparisons PCa M1 vs. controls or PCa M1 vs. PCa M0. **d)** Genomic annotation (location within 3' or 5'UTR, distal intergenic region, downstream region, exon or intron, promotor region) and assessment of CpG-associated landscapes (island, open sea, shelf, shore) within common significant, hypermethylated DMRs (logFC > 1) in plasma cfDNA between the comparisons PCa M1 vs. controls or PCa M1 vs. PCa M0. **e)** Genomic annotation (location within 3' or 5'UTR, distal intergenic region, downstream region, exon or intron, promotor region) and assessment of CpG-associated landscapes (island, open sea, shelf, shore) within common significant, hypomethylated DMRs (logFC < 0) in plasma cfDNA between the comparisons PCa M1 vs. controls or PCa M1 vs. PCa M0. **f)** Common significant DMRs in urinary cfDNA between the comparisons PCa M1 vs. controls or PCa M1 vs. PCa M0. **g)** Genomic annotation (location within 3' or 5'UTR, distal intergenic region, downstream region, exon or intron, promotor region) and assessment of CpG-associated landscapes (island, open sea, shelf, shore) within common significant DMRs in urinary cfDNA between the comparisons PCa M1 vs. controls or PCa M1 vs. PCa M0. Abbreviations: cfDNA = cell-free DNA, DMRs = differentially methylated regions, logFC = log fold change, M0/M1 = absence/presence of distant metastases, n = number, PCa = prostate cancer, UTR = untranslated region

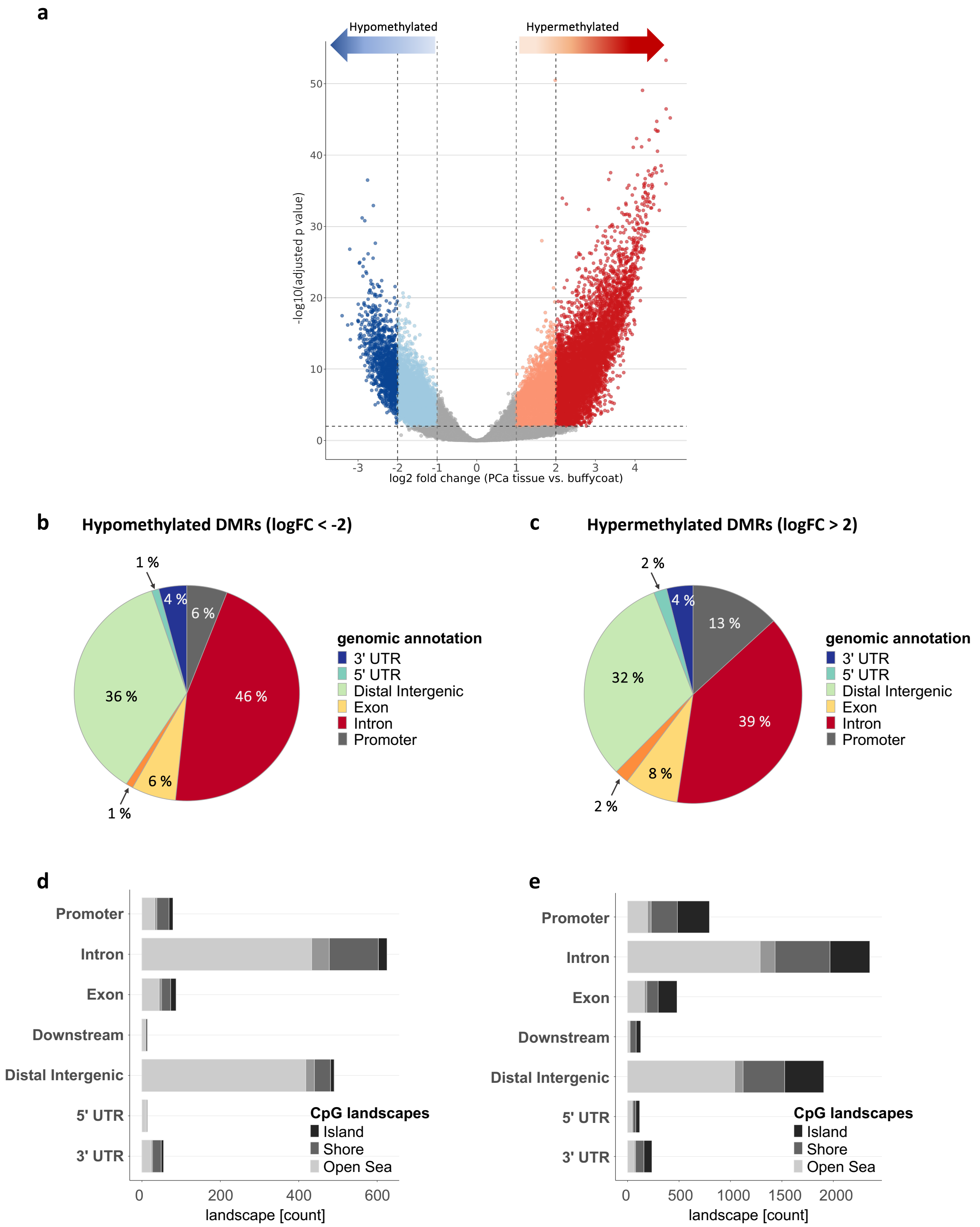

Supplementary Figure S2: Genome-wide methylation profiling in genomic DNA from PCa tissue. **a)** Overview of the results from the DMR analysis between eight PCa tissue and matched buffy coat samples. Distribution of log2-fold changes (log2FC) and adjusted p values in 810,174 genomic regions. Correction for multiple testing was performed with Benjamini-Hochberg method, significance was defined as adjusted p value < 0.01. Vertical dashed lines: log2FC = +- 1 and +-2, respectively; horizontal dashed line: adjusted p value = 0.01. **b)** Genomic annotation (location within 3' or 5'UTR, distal intergenic region, downstream region, exon or intron, promotor region) of 1364 significant, hypomethylated DMRs (logFC < -2) in PCa tissue vs. buffy coat. **c)** Genomic annotation (location within 3' or 5'UTR, distal intergenic region, downstream region, exon or intron, promotor region) of 6015 significant, hypermethylated DMRs (logFC > 2) in PCa tissue vs. buffy coat. **d)** Assessment of CpG-associated landscapes (island, open sea, shelf, shore) in 1364 significant, hypomethylated DMRs (logFC < -2) in PCa tissue vs. buffy coat. **e)** Assessment of CpG-associated landscapes (island, open sea, shelf, shore) in 6015 significant, hypermethylated DMRs (logFC > 2) in PCa tissue vs. buffy coat.

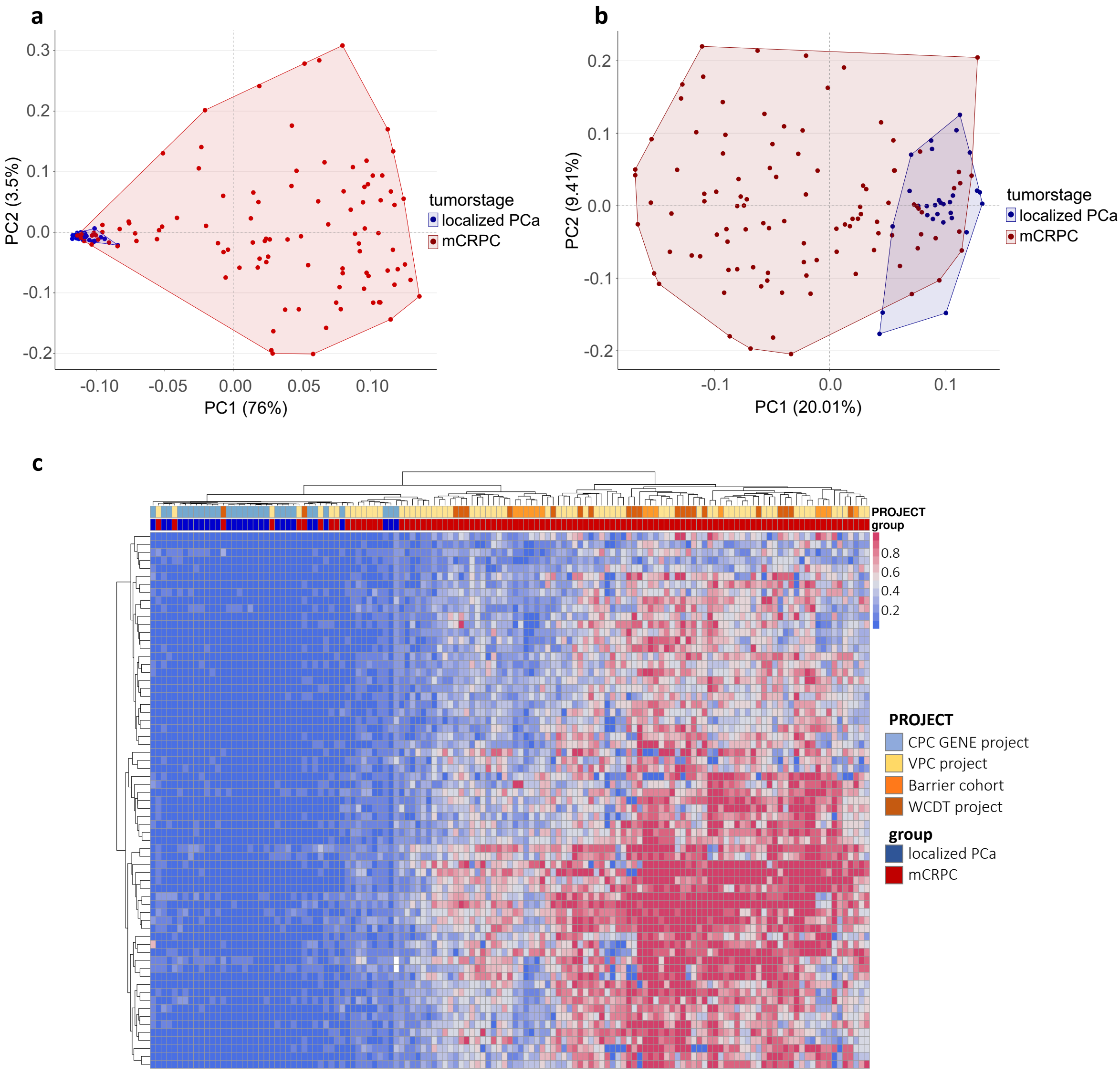

Supplementary Figure S3: Clustering based on β-values in 67 marker regions for plasma samples from lPCa or mCRPC patients from an external cohort (Toronto, Chen et al.[1]). **a)** PCA based on β-values in the 67 marker regions. **b)** PCA based on β-values in 67 randomly selected, genome-wide 300bp-regions with similar CpG density (CpG density >6) compared to the 67 marker regions from own tissue analysis. **(a+b)** X- and y-axes display the first and second component, explaining the most and second most proportion of the variance, respectively, with the respective proportions (%) reported next to the axis title. **c)** Hierarchical clustering based on β-values in the 67 marker regions. The project names were derived from the original publication[1]. Abbreviations: lPCa = localized prostate cancer, mCRPC = metastatic castration-resistant prostate cancer, PCA = principal component analysis

[1] Chen, S. *et al.* The cell-free DNA methylome captures distinctions between localized and metastatic prostate tumors. *Nat. Commun.* **13**, 6467 (2022). https://doi.org/10.1038/s41467-022-34012-2

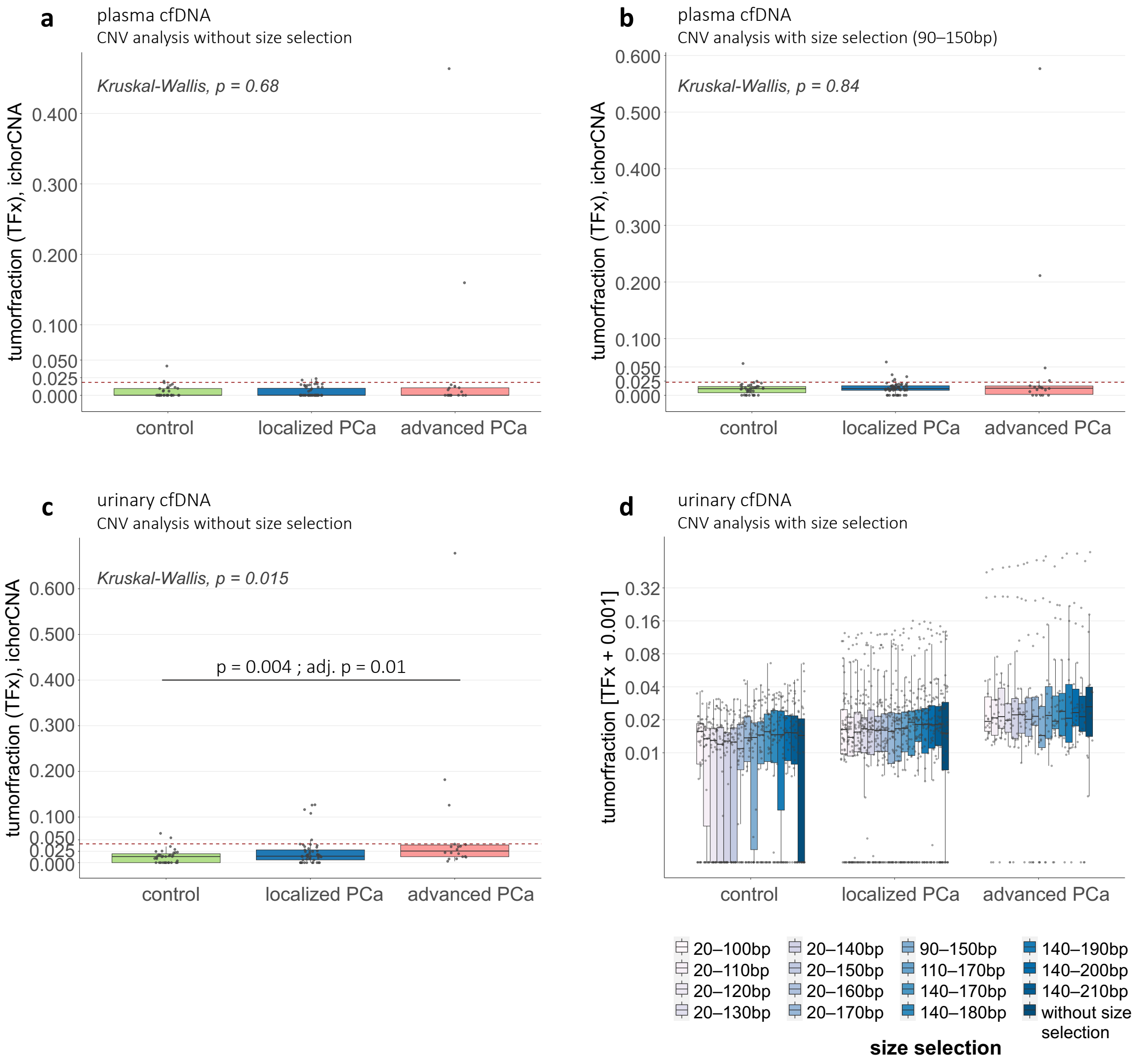

Supplementary Figure S4: Genome-wide assessment of CNVs in plasma and urinary cfDNA. **a)** Tumor fractions estimated with the ichorCNA algorithm based on the CNV analysis without size selection in plasma samples from cancer-free controls, lPCa patients, and aPCa patients. **b)** Tumor fractions estimated with the ichorCNA algorithm based on the CNV analysis after in-silico size selection for cfDNA fragments with 90–150bp lengths in plasma samples from cancer-free controls, lPCa patients, and aPCa patients. **c)** Tumor fractions estimated with the ichorCNA algorithm based on the CNV analysis without size selection in urine samples from cancer-free controls, lPCa patients, and aPCa patients. **(a–c)** The horizontal, red dotted line represents the ctDNA detectability threshold (95th percentile of control cohort). Results between the three cohorts were statistically compared with Kruskal-Wallis testing, significant results were defined as p value <0.05. **d)** Tumor fractions estimated with the ichorCNA algorithm based on the CNV analysis after in-silico size selection for different cfDNA fragment length ranges in urine samples from cancer-free controls, lPCa patients, and aPCa patients. Log2-transformed y-axis; TFx with pseudo count (0.001). **(a–d)** Box plot center lines indicate the median, and boxes illustrate the interquartile range with Tukey whiskers. Each dot represents one sample. Abbreviations: bp = base pairs, CNVs = copy number variations, lcWGS = low-coverage whole-genome sequencing, TFx = tumor fraction

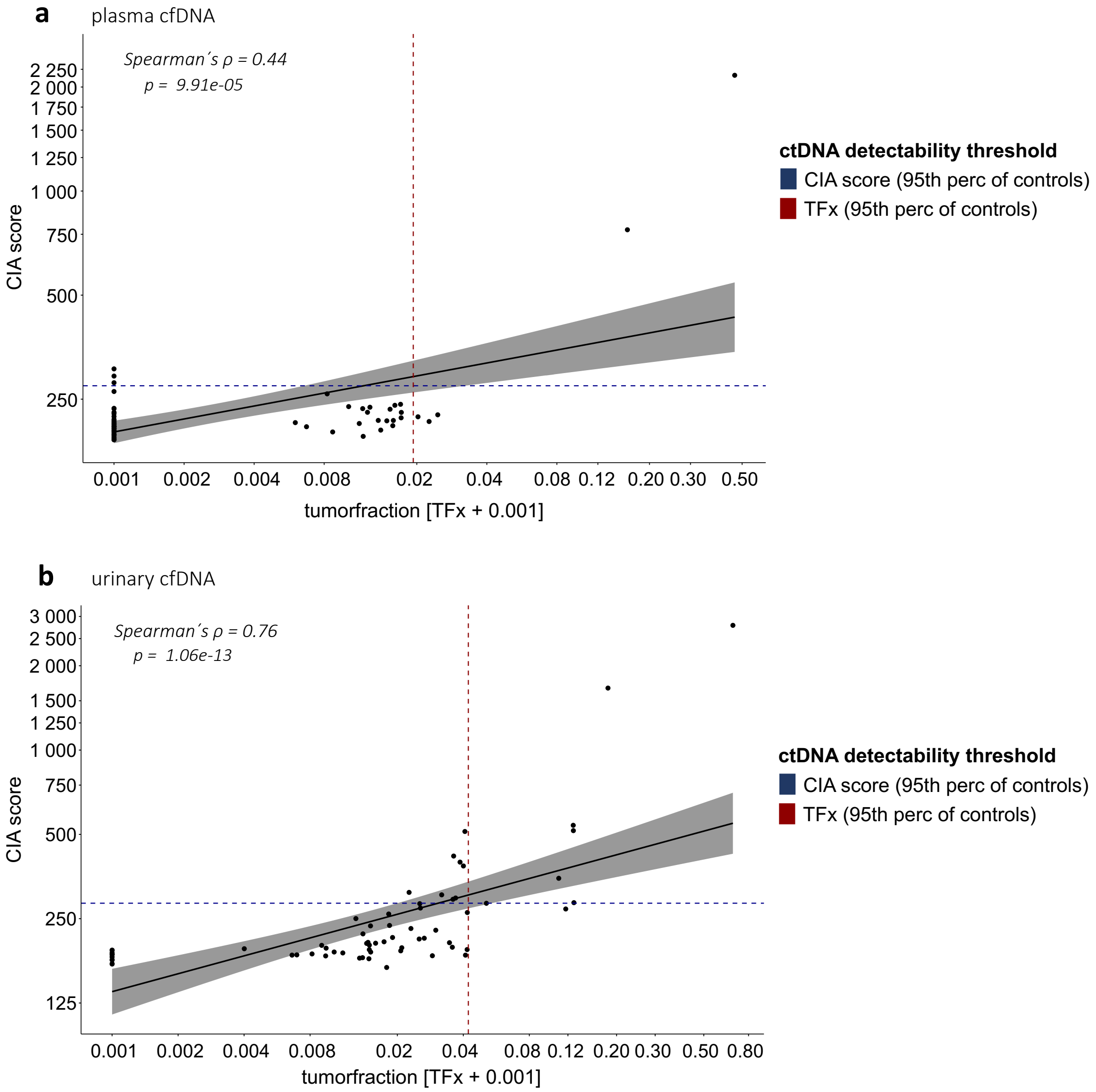

Supplementary Figure S5: Correlation between genomic biomarkers in plasma and urinary cfDNA. Spearman correlation between estimated TFx based on CNV analysis with ichorCNA (x-axis) and CIA score (y-axis) for all plasma **a)** and urine **b)** samples from PCa patients. Each dot represents one sample. Both CIA score and TFx are log_2-transformed. The blue dotted line represents the 95th percentile of the CIA score from controls, while the red dotted line denotes the 95th percentile of TFx from controls, both serving as the respective ctDNA positivity thresholds. Abbreviations: CIA = chromosomal instability analysis

a

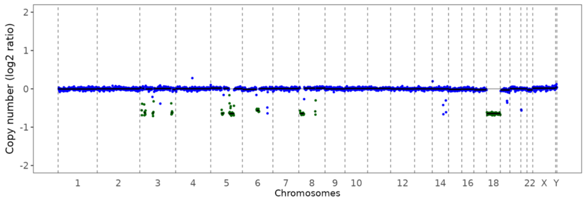

Gleason 7a ; pT2c pNx (cN0 cM0) L0 V0 Pn1;
80% tumor cell content

b

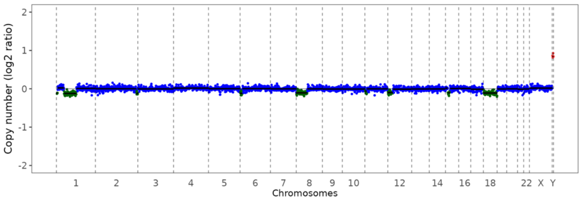

Gleason 7a; pT3b pN1 (1/21) cM0 L1 V0 Pn1;
60% tumor cell content

c

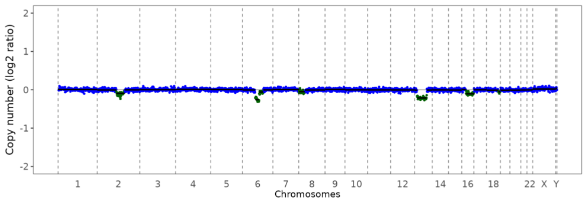

Gleason 7a; pT2c pNx (cN0 cM0) L0 V0 Pn1;
70% tumor cell content

d

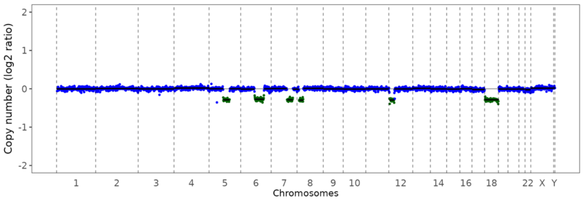

Gleason 7a; pT3a pNx (cN0 cM0) L0 V0 Pn1;
70% tumor cell content

e

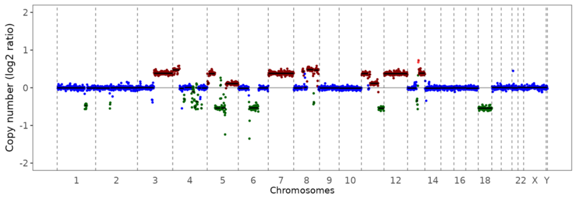

Gleason 9; pT3b pN1 (2/22) cM0 L1 V0 Pn1;
70% tumor cell content

f

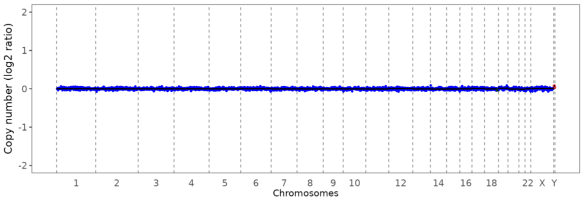

Gleason 7a; pT3b pNx (cN0 cM0) V0 Pn0;
50% tumor cell content

g

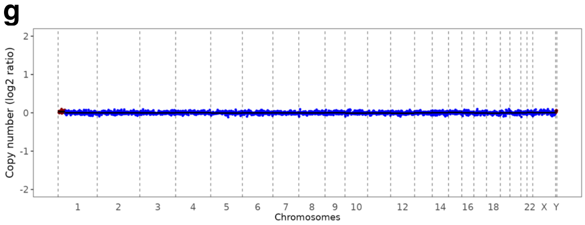

Gleason 7a; pT3a pN0 (0/8) cM0 L1 V0 Pn1;
90% tumor cell content

h

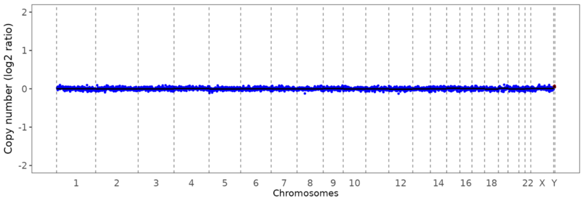

Gleason 7a; pT3a pNx (cN0 cM0) L0 V0 Pn1;
40% tumor cell content

i

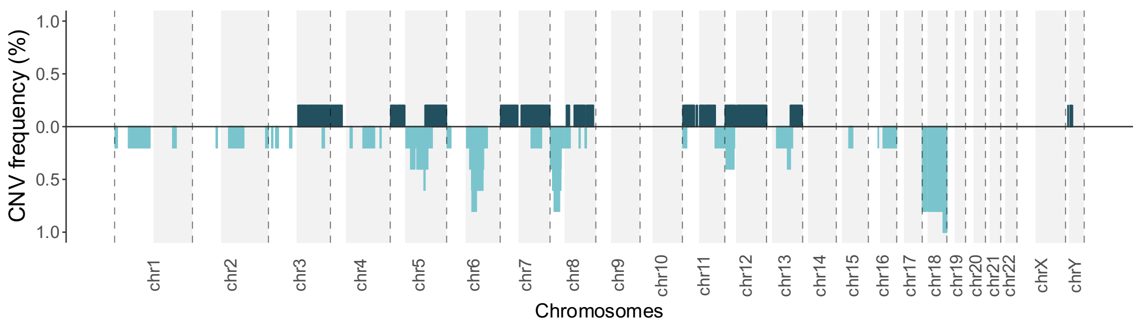

Supplementary Figure S6: Genome-wide assessment of CNVs in genomic DNA from fresh-frozen PCa tissue samples. **(a–h)** Single CNV profiles of eight PCa tissue samples. **(a–e)** PCa tissue samples with detectable alterations. **(f–h)** PCa tissue samples without detectable alterations. **i)** Summary of recurrent amplifications and deletions in 5 PCa tissue samples with detectable CNVs and TFx >10%. The y-axis indicates the frequency of a

detected copy number state at the chromosomal coordinate specified on the x-axis across the samples. Areas shaded in gray represent the q-arm of the respective chromosome. Abbreviations: chr = chromosome, cM = clinical assessment of distant metastases, cN/pN = clinical/pathological assessment of lymph node metastases, L0/L1 = absence/presence of lymphatic invasion, N0/N1 = absence/presence of lymph node metastases, Nx = unknown lymph node status, V0 = no venous invasion, Pn0/Pn1 = absence/presence of perineural invasion, pT = pathological assessment of the primary tumor's extent

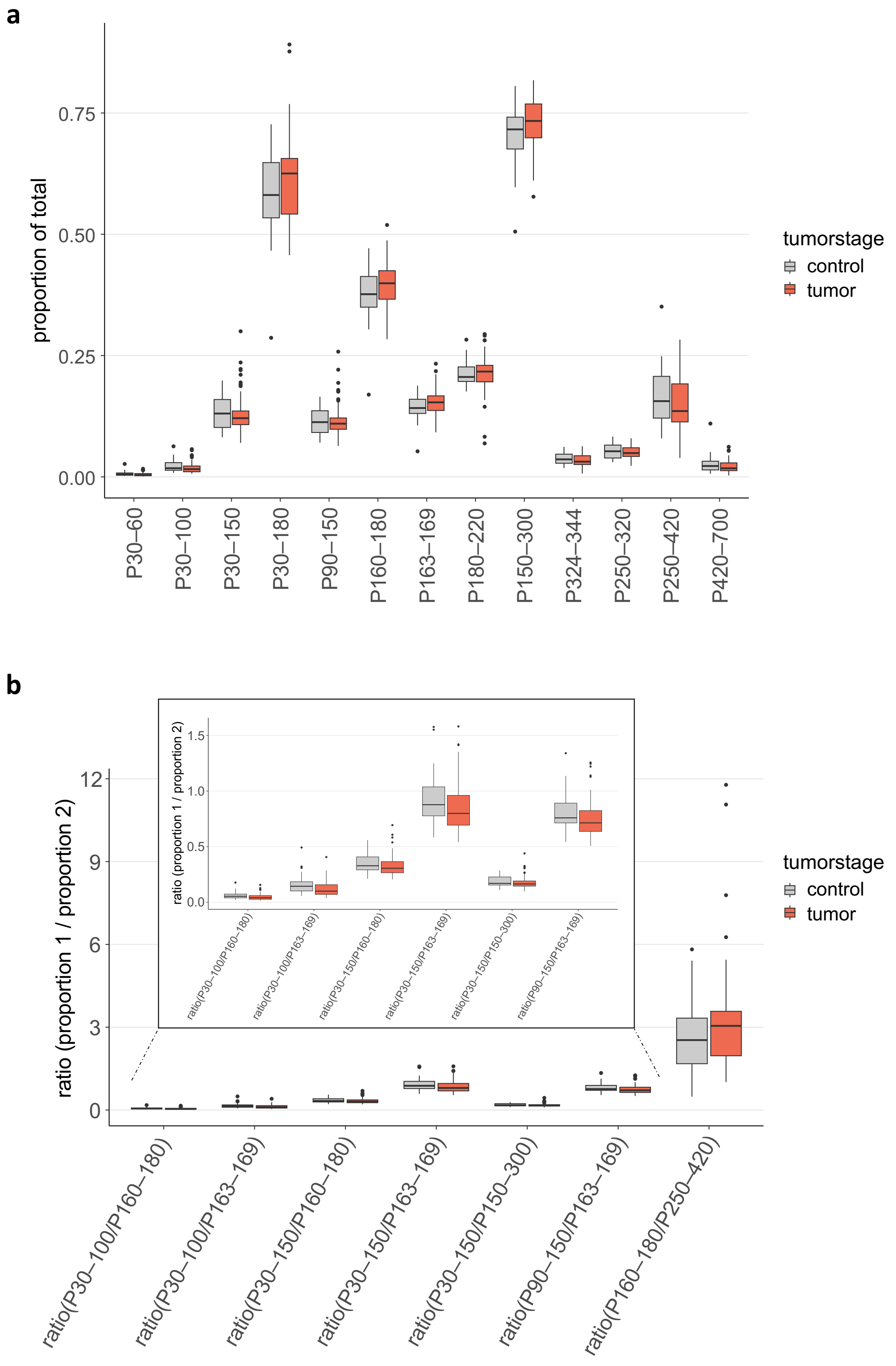

Supplementary Figure S7: Plasma cfDNA fragmentation analysis. **a)** Different proportions of plasma cfDNA fragment length ranges (in relation to all fragments with 30–700 bp length) in all tumor and control samples. **b)** Different ratios of proportions of plasma cfDNA fragment length ranges (in relation to all fragments with 30–700 bp length) in all tumor and control samples. The additional box displays a zoomed window. **(a+b)** Box plot center lines indicate the median, and boxes illustrate the interquartile range with Tukey whiskers. Dots represent outlying samples.

a

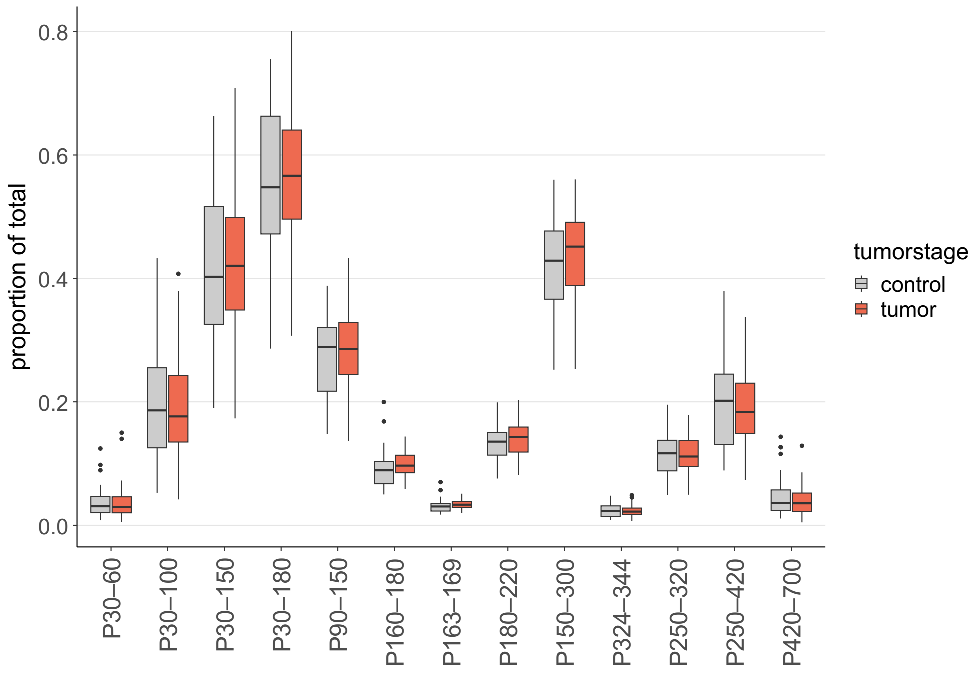

b

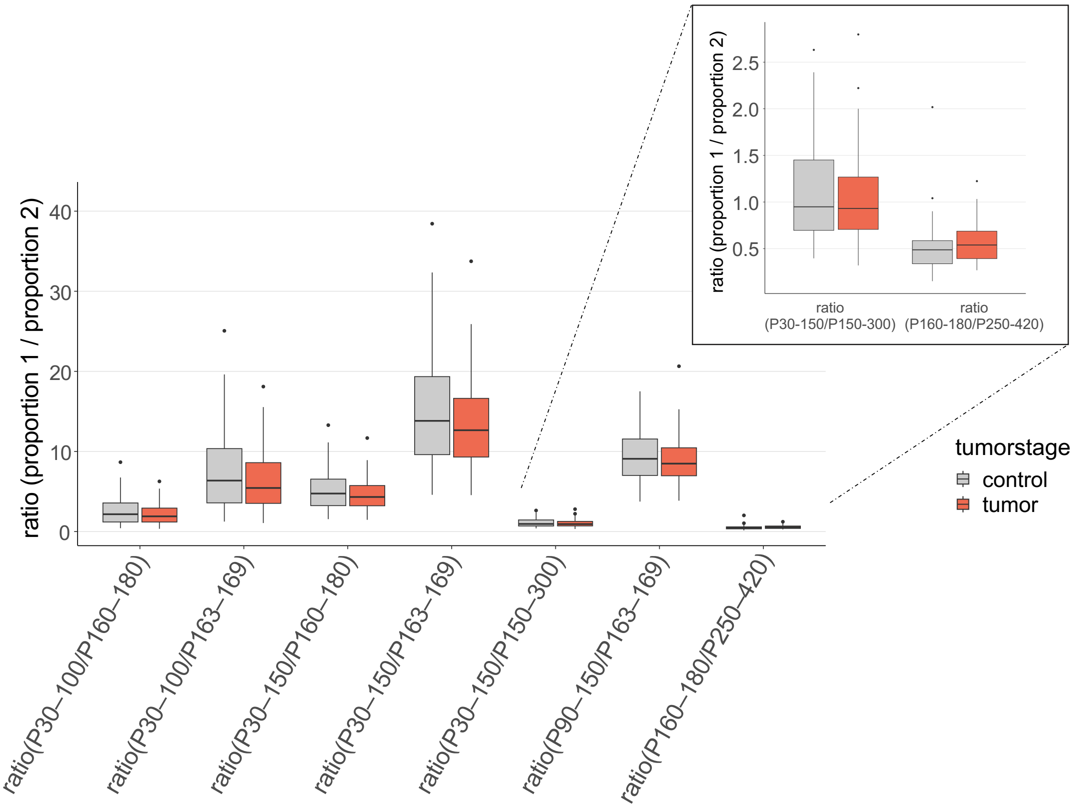

Supplementary Figure S8: Urinary cfDNA fragmentation analysis. **a)** Different proportions of urinary cfDNA fragment length ranges (in relation to all fragments with 30–700 bp length) in all tumor and control samples. **b)** Different ratios of proportions of urinary cfDNA fragment length ranges (in relation to all fragments with 30–700 bp length) in all tumor and control samples. The additional box displays a zoomed window. **(a+b)** Box plot center lines indicate the median, and boxes illustrate the interquartile range with Tukey whiskers. Dots represent outlying samples.

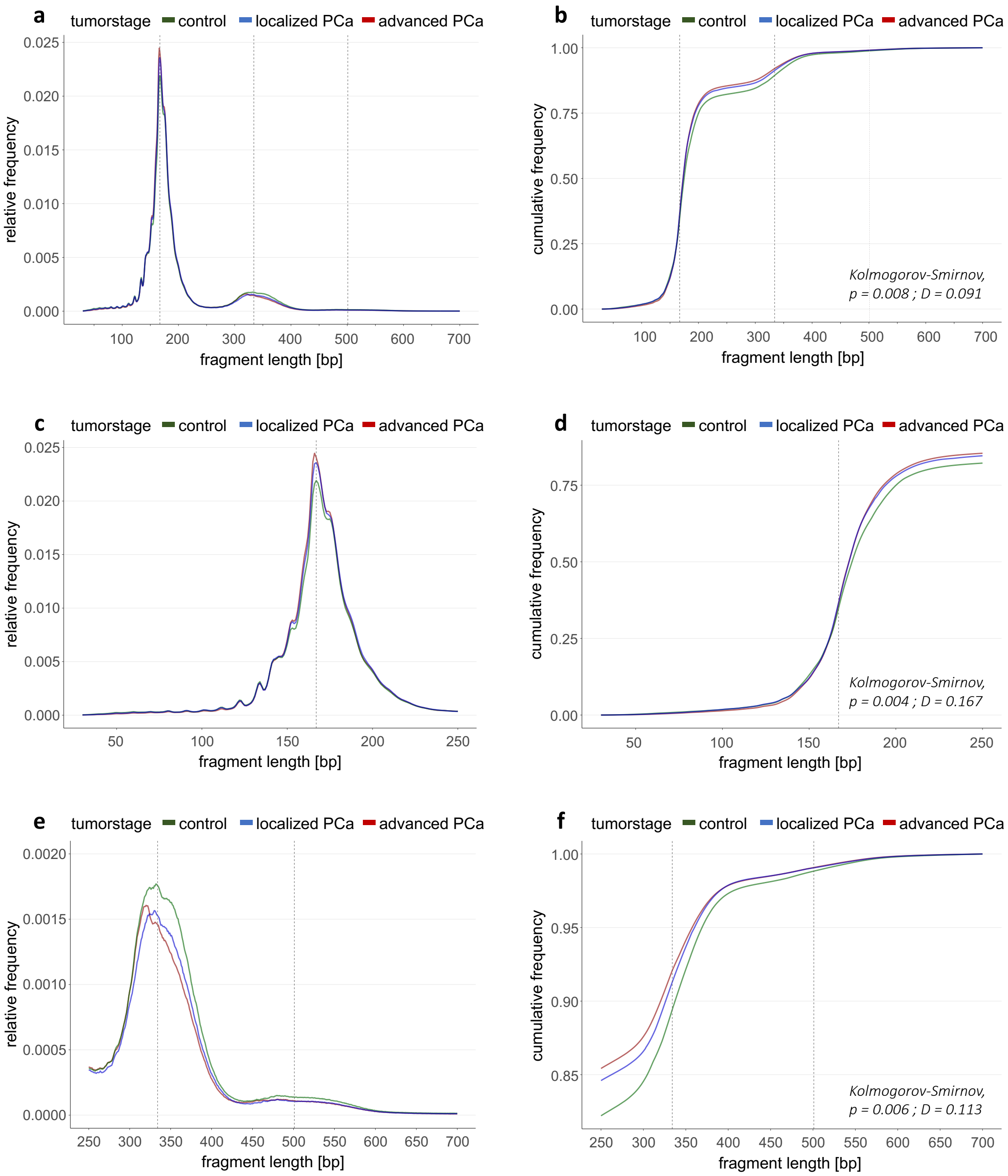

Supplementary Figure S9: Relative and cumulative frequency distributions of plasma cfDNA fragmentation. **(a–f)** Plasma cfDNA fragmentation profiles represented as median profiles of all samples from lPCa and aPCa patients, and cancer-free controls. **(a+b)** Distribution within fragment length range 30–700 bp. **(c+d)** Distribution within fragment length range 30–250 bp. **(e+f)** Distribution within fragment length range 250–700 bp. Y-axis: **(a, c, e)** relative frequencies of cfDNA fragments with specific length (bp) compared to all fragments (30–700 bp fragment length) and **(b, d, f)** cumulative frequencies. Vertical dotted grey line(s) indicate 167 bp and its multiples, 334 bp (2 x 167 bp) and 501 bp (3 x 167 bp). Median cumulative distributions between all tumor samples and control samples were compared with Kolmogorov-Smirnov testing. D = distance (D statistic)

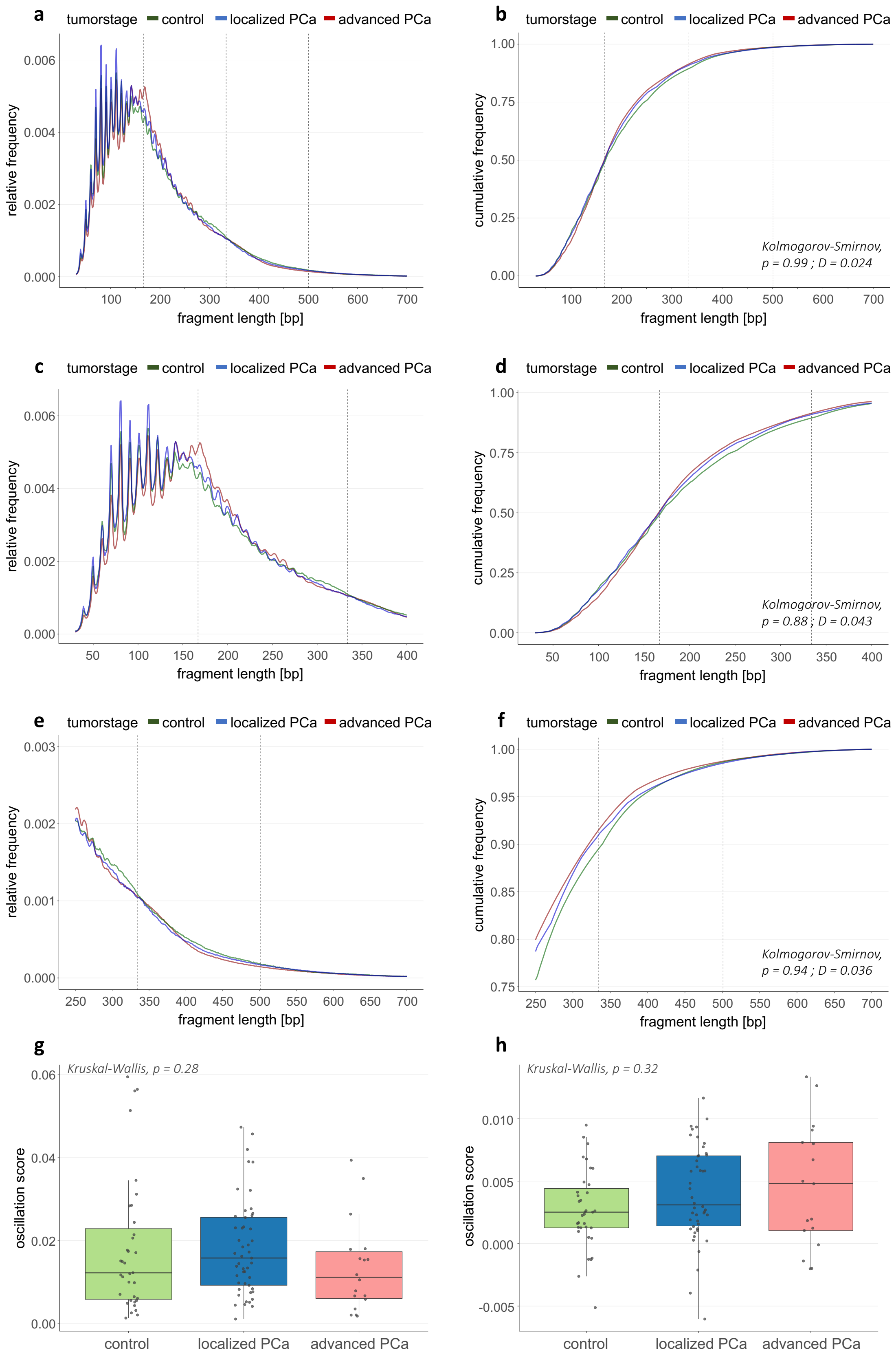

Supplementary Figure S10: Relative and cumulative frequency distributions of urinary cfDNA fragmentation. **(a–f)** Urinary cfDNA fragmentation profiles represented as median profiles of all samples from lPCa and aPCa patients, and cancer-free controls. **(a+b)** Distribution within fragment length range 30–700 bp. **(c+d)** Distribution

within fragment length range 30–400 bp. **(e+f)** Distribution within fragment length range 250–700 bp. Y-axis: **(a, c, e)** relative frequencies of cfDNA fragments with specific length (bp) compared to all fragments (30–700 bp fragment length) and **(b, d, f)** cumulative frequencies. Vertical dotted grey line(s) indicate 167 bp and its multiples, 334 bp (2 x 167 bp) and 501 bp (3 x 167 bp). Median cumulative distributions between all tumor samples and control samples were compared with Kolmogorov-Smirnov testing. **g)** 10bp-oscillation scores in urinary cfDNA fragments with 30–150 bp fragment length from cancer-free controls, lPCa patients, and aPCa patients. **h)** 10bp-oscillation scores in urinary cfDNA fragments with 150–300 bp fragment length from cancer-free controls, lPCa patients, and aPCa patients. **(g+h)** Box plot center lines indicate the median, and boxes illustrate the interquartile range with Tukey whiskers. Each dot represents one sample. Results between the three cohorts were statistically compared with Kruskal-Wallis testing, significant results were defined as p value < 0.05.

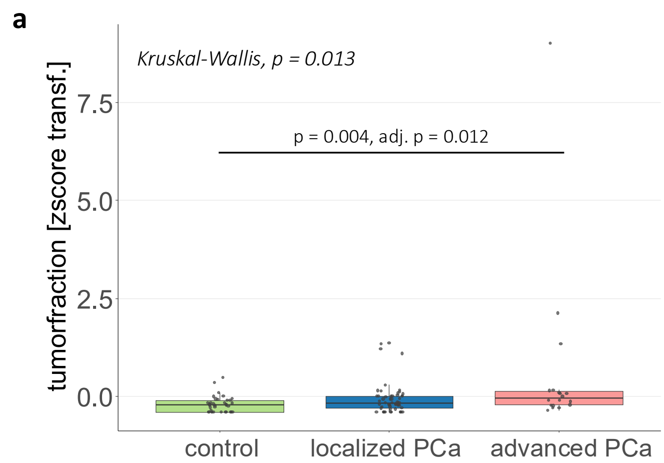

	plasma	urine
localized PCa	30 (55%)	25 (45%)
advanced PCa	6 (33%)	12 (67%)

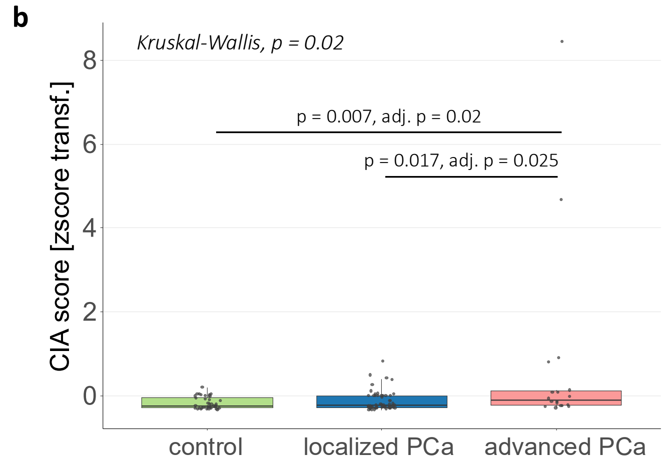

	plasma	urine
localized PCa	37 (67%)	18 (33%)
advanced PCa	7 (39%)	11 (61%)

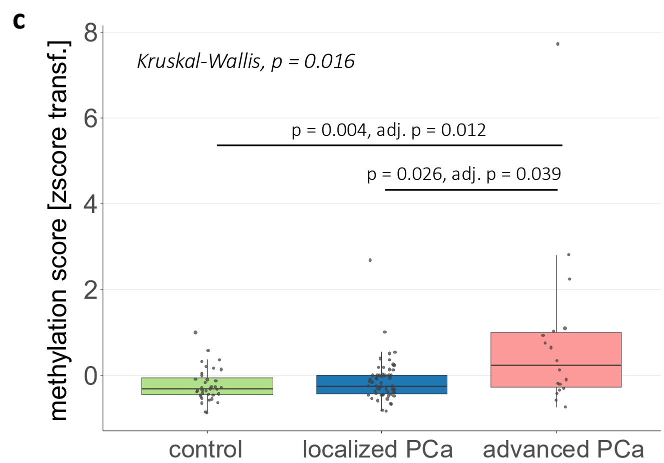

	plasma	urine
localized PCa	34 (61%)	21 (38%)
advanced PCa	8 (38%)	10 (56%)

Supplementary Figure S11: Comparison of highest per-patient cfDNA feature values across controls, lPCa, and aPCa patients. For each patient, the higher of the z-score–transformed values for TFx, CIA score and methylation score between matched plasma and urine samples was used to capture the strongest molecular signal across biofluids. Box plots show the distributions of **a)** estimated TFx, **b)** CIA score, and **c)** methylation score in controls, lPCa, and aPCa patients. Box plot center lines indicate the median, and boxes illustrate the interquartile range with Tukey whiskers. Each dot represents one patient. The three cohorts were compared using Kruskal-Wallis testing, followed by Dunn's post hoc test. Adjustment for multiple testing was performed with Benjamini Hochberg´s method. Only significant differences are shown. Tables adjacent to each plot indicate the proportion of patients in whom the highest signal was detected in plasma or urine, respectively.
